# Dissecting The role of *Plasmodium* metacaspase-2 in malaria gametogenesis and sporogony

**DOI:** 10.1080/22221751.2022.2052357

**Published:** 2022-03-30

**Authors:** Vandana Kumari, Kona Madhavinadha Prasad, Inderjeet Kalia, Gagandeep Sindhu, Rajnikant Dixit, Diwan S. Rawat, O. P. Singh, Agam P. Singh, Kailash C. Pandey

**Affiliations:** a ICMR-National Institute of Malaria Research, New Delhi, India; b National Institute of Immunology, New Delhi, India; c Depatment of Chemistry, University of Delhi, New Delhi, India; d Academy of Scientific and Innovative Research (AcSIR), Ghaziabad Uttar Pradesh, UP, India

**Keywords:** Malaria, metacaspase, specific inhibitor, *Plasmodium* transmission, sporogony, *An. stephensi*, gametogenesis, ookinete

## Abstract

The family of apicomplexan specific proteins contains caspases–like proteins called “metacaspases”. These enzymes are present in the malaria parasite but absent in human; therefore, these can be explored as potential drug targets. We deleted the MCA-2 gene from *Plasmodium berghei* genome using a gene knockout strategy to decipher its precise function. This study has identified that MCA-2 plays an important role in parasite transmission since it is critical for the formation of gametocytes and for maintaining an appropriate number of infectious sporozoites required for sporogony. It is noticeable that a significant reduction in gametocyte, oocysts, ookinete and sporozoites load along with a delay in hepatocytes invasion were observed in the MCA-2 knockout parasite. Furthermore, a study found the two MCA-2 inhibitory molecules known as C-532 and C-533, which remarkably inhibited the MCA-2 activity, abolished the *in vitro* parasite growth, and also impaired the transmission cycle of *P. falciparum* and *P. berghei* in *An. stephensi*. Our findings indicate that the deletion of MCA-2 hampers the *Plasmodium* development during erythrocytic and exo-erythrocytic stages, and its inhibition by C-532 and C-533 critically affects the malaria transmission biology.

## Highlights

- Deletion of Metacaspase-2 (MCA-2) caused an unregulated stress-mediated apoptosis-like cell death and reduced the formation of gametocytes.
- A significant reduction in oocysts, ookinete and sporozoites load, and delay in hepatocytes invasion in the Δ*Pb*MCA-2 parasites.
- MCA-2 inhibitory molecule, C-532 and C-533, inhibits *Pf*MCA-2 activity along with the parasite asexual and sexual growth *in vitro* and *in vivo*.
- C-532 and C-533 impair *Plasmodium* transmission in *An. stephensi*, possibly by inhibiting MCA-2.

## Introduction

The growth and development of the malaria parasite in the vertebrate host is a complex process, and several host and parasite proteases are involved in this process. Beginning from the bite by an infected female *Anopheles* mosquito, *Plasmodium* sporozoites reach the liver, where they invade hepatocytes. The parasite either lies dormant or develops over several days, eventually forming the liver-schizonts that prelude to a blood-stage infection. During the course of infection, several parasite proteases are involved in the different stages of the parasite, and many are well-characterized for their role in *Plasmodium* pathogenesis. For instance, Falcipains and plasmepsins-I-IV degrade host hemoglobin for parasite nourishment [1–3]; Plasmepsin-V, IX and X are involved in the trafficking pathway [4], invasion and egress, respectively [5]. Furthermore, Plasmepsin-VIII was essential for sporozoites gliding and mortality [6]. Several proteases played a crucial role in the completion of parasite sexual development. For example, *Pf*PH, a protein expressed in sexual stages; important for the transition of parasite from zygote to oocysts [7]; a transcription factor, *Pf*AP2-G was also known as a major cell fate determinant that triggers the sexual commitment of parasite by activating genes required for early gametocytogenesis [8]. Similarly, *Pfs*230, *Pfs*48/45, and *Pfs*25, were known markers for sexual development of the parasite [9–11]. Considering the importance of parasite proteases, we explored the *Plasmodium* database and came across with partially characterized proteases termed as “Metacaspases”. The genome of the malaria parasite *Plasmodium* encodes for three metacaspases genes [12].

Metacaspases are cysteine-dependent proteases sharing the conserved His-Cys catalytic dyad of caspases [13]. These proteases are members of the C14 family, clan CD, with a difference in substrate specificity compared to caspases [14]. In addition, metacaspases have a highly acidic S1 pocket leading to arginine and lysine substrate specificity at the P1 position, compared to the aspartic acid specificity reported for caspases [13,15]. Previous studies showed that *P. falciparum* metacaspase-1 (*Pf*MCA-1) was probably responsible for cell death in response to chloroquine treatment *in vitro* and caused ookinete growth arrest [16–18]. Furthermore, *Pf*MCA-1 possess the His-Cys catalytic dyad and upstream signalling pathways such as death domain or CARD, a module of 90–100 amino acids involved in apoptosis signalling pathways [17,18]. Similarly, *Pf*MCA-3 was an unusual serine-like protease, lacking His-Cys catalytic dyad and predominantly expressed in the asexual stages [17]. However, the functional importance of *Pf*MCA-3 remains unclear. Our earlier report suggested that *Pf*MCA-2 possess His-Cys catalytic dyad and its inhibition by its specific inhibitor, SS-5, causes stress-dependant apoptosis-like cell death [19]. An earlier study revealed that *Pf*MCA-2 is uniformly expressed in the blood stage (schizont) and sexual stages (gametocyte stage-I to IV), but its role during the parasite exo-erythrocytic cycle has not been elucidated [20].

In the present study, we have characterized the MCA-2 in terms of its detailed function in the parasite sporogony and transmission cycle by genetic knockout and using inhibitors, C-532 & C-533.

## Material and methods

### Ethical statement

Malaria parasite bank of ICMR-National Institute of Malaria Research (ICMR-NIMR), New Delhi, has been approved by the Institutional Ethical Committee for *in vitro* culture of *P.falciparum*. The animal experiments were performed as per the protocols approved by the Institutional Animal Ethics Committee (IAEC) of National Institute of Immunology and ICMR-NIMR, under the sanctioned number NII/IAEC/483/18 and IAEC/NIMR/2019-1/09, respectively. The Committee for Control and Supervision of Experiments on Animals (CPCSEA) is a statutory body formed by the Act of the Indian Parliament under the Prevention of Cruelty to Animals Act 1960. This statutory body is under the Ministry of Environment, Forest and Climate change, and CPCSEA comes under the wildlife division of the ministry (http://moef.gov.in/wildlife). The guidelines followed for experiments are as described on the website (http://cpcsea.nic.in/Content/55_1_GUIDELINES.aspx).

### Generation of PbMCA-2 knockout line

*Pb*MCA-2 knockout (Δ*Pb*MCA-2) parasites were generated by a double homologous recombination method. The *P. berghei*-ANKA wild-type parasites were used to generate Δ*Pb*MCA-2 [*Pb*Δ*Pb*MCA-2: Hdhfr & GFP]. In order to generate the *Pb*MCA-2 knockout-targeting construct, a 799 bp fragment was amplified from the 5’ UTR region of *Pb*MCA-2 (gene id: PBANKA_1302300, primers *Pb*MCA-2-5U-XhoI Fw, and *Pb*MCA-2-5U ClaI Rev) and a 741 bp from the 3’UTR of the gene (primers *Pb*MCA-2-3U-NotI Fw and *Pb*MCA-2-3U-AscI Rev). Both UTRs were cloned in pBC derived knockout vector flaking the GFP and DHFR cassette. The details of the primers are summarized in Table.1. The targeting construct DNA was isolated by using an Endo-Free Plasmid purification kit (Invitrogen, Carlsbad, CA). The construct DNA was further linearized with ScaI restriction enzyme, and the linearized plasmid was electroporated into *P. berghei*-ANKA schizonts using an Amaxa nucleo-factor device (LONZA). The electroporated parasites were intravenously injected into C57BL/6 mice. Upon the appearance of parasites, mice were treated with pyrimethamine (10 mg/kg of body weight) for 3–4 days [21–23].

### Validation of transgenic parasite line

Genotyping of the transformed parasites was done by diagnostic PCR and Southern blot analysis. Parasite genomic DNA was isolated using MDI genomic DNA Mini-prep kit (MDI, India) and analyzed by diagnostic PCR using the following set of primers, Dig-Fw, (hDHFR–3'UTR) and Dig-Rev, (*Pb*MCA-2-3'UTR) in order to confirm correct integration. The transgenic parasites were also screened for GFP signals by live-cell fluorescence microscopy, Immunofluoresence assay (IFA) and western blotting with MCA-2 antibody. The mixed population of transgenic parasites were then subjected to dilution cloning in order to obtain a single parasite clonal line. For Southern blot analysis, genomic DNA of positive clones was isolated, digested with EcoRI for 3 hrs, and run on gel at 25 V for 14 hrs. The southern transfer was carried out overnight by the capillary action to a positively charged nylon membrane (Roche). The transferred DNA was UV cross-linked using a charge of 120 mJ to the nylon membrane followed by blotting using DIG-High Prime DNA Labelling and Detection Starter Kit II from Roche (Cat# 11585614910) as per the manufacturer's protocol [22,23].

## Blood-stage characterization

### Phenotype and growth profile of ΔPbMCA-2

An equal number of the parasite, approx. 1 × 10^7^ cells were further injected into three groups of animals; Δ*Pb*MCA-2-c1 (i), Δ*Pb*MCA-2-c4 (ii), and wild *P. berghei* ANKA (iii), containing four mice in each group. Thin blood smears were made daily from the infected mouse's tail vein up to 5–6 days [23]. The no. of parasites in each group of animals was calculated by SYBR green–I staining. Infected blood was centrifuged and passed through the CF 11 cellulose column to remove the plasma and buffy coat. Once WBCs removed, 200 µl culture was plated in 96-well plate in triplicate. Cultures were centrifuged (1200 rpm, 5 min) and washed twice with 100 µl 1X phosphate-buffer saline (PBS) + 0.5% bovine serum albumin (BSA) + 0.02% sodium azide. Cells were then incubated with 75 µl of SYBR Green I dye (1:1000) (Invitrogen, Carlsbad, CA) for 20-40 min at 25°C in the dark. The fluorescence intensity was measured at 485 nm excitation and 528 nm emissions using a fluoro-spectrophotometer [24].

### Measurement of cell viability and reactive oxygen species (ROS)

To examine the cell viability in Δ*Pb*MCA-2 parasites, 200µl mixed stage parasites without WBCs were plated in 96-well plate in triplicates followed by the addition of 10µl of AlamarBlue (Invitrogen, Carlsbad, CA). Parasites viability was monitored at different time points (2, 4, 8, 12 hrs) using AlamarBlue dye [25,26]. Wells containing an equivalent number of uninfected RBCs were also tested. Similarly, mixed stage parasites were washed and resuspended in RPMI followed by incubation with a cell-permeant dye, H2DCFDA (Sigma), for 30 min [25,27]. Fluorometric measurements (excitation at 510 nm and emission at 530 nm) were performed in duplicate, and the results were expressed as the mean fluorescence intensity [25].

### Measurement of mitochondrial transmembrane potential [Δψm]

The mitochondrial transmembrane potential was investigated using JC-1 dye. JC-1 accumulates in the mitochondrial matrix under the influence of Δψm, where it reversibly forms monomers (green) with characteristic absorption and emission spectra [27,28]. Briefly, Δ*Pb*MCA-2 mixed stages parasites cells were incubated with JC-1 dye at 5µg/µl concentration. The reactions were then analyzed by fluorescence measurement using a spectrofluorometer at 507 and 530 nm as the excitation and emission wavelengths, respectively (green fluorescence), and at 507 and 590 nm as excitation and emission wavelengths, respectively (red fluorescence). The ratio of the reading at 590 nm to the reading at 530 nm (590:530 ratios) was considered the relative Δψm value. In flow cytometry, the FL-1 channel denotes the mean fluorescence intensity [27].

### Measurement of intracellular Ca^2+^ and ATP level

Intracellular calcium (Ca^2+^) concentration was measured with the fluorescent probe fura-2-acetoxymethyl ester (fura-2AM), as described previously [28]. Briefly, Δ*Pb*MCA-2 mixed stages parasites were harvested and incubated with fura-2 AM (6µM) at 37°C for 30–60 min [20,27,28]. Similarly, ATP content was determined by the luciferin/luciferase method, as described previously [28]. In brief, Δ*Pb*MCA-2 and *P. berghei* ANKA mixed stages of parasites were washed with 1x PBS twice, and the cytosolic extract was prepared as described earlier. An aliquot of the lysate was assayed for ATP using the luciferase ATP assay kit (Invitrogen, Inc. Ltd.). The amount of ATP in the experimental samples was calculated from a standard curve prepared with ATP.

### Flow cytometry analysis

For flow cytometry analysis, 200µl of Δ*Pb*MCA-2 (mixed stage culture) and *P. berghei* ANKA parasites were seeded in the 96-well plate. Briefly, infected RBCs cells were washed and fixed with 1% p-formaldehyde/1x PBS on ice for 30 min followed by permeabilization with 0.1% Triton X-100 in 0.1% sodium citrate for 20 min on ice. The TdT enzyme labelling was done, followed by flow cytometry. For microscopy, TdT enzyme stained cells were deposited onto slides prior to the AlexaFluor488 treatment and slides were observed under a fluorescence microscope after staining with DAPI. The unlabelled parasites were used as staining controls [20,30,31]. Similarly, approximately 1 × 10^7^ parasitized RBC (pRBC) cells along with uninfected RBC treated with 0.05% saponin and were washed with 1x PBS and suspended in 100 µl of 1x annexin binding buffer and stained with annexin-FITC and propidium iodide (PI) for 15-20 min in the dark according to manufacturer's instructions (Annexin V- FITC Assay kit, Cayman, USA). The experiment was performed in triplicates individually. Flow cytometry analysis was performed by a FACS Calibur flow cytometry, and data was analyzed by BD FACScan software (BD Biosciences, US) [29–33].

### Drug sensitivity assay

Twenty-two BALBc mice weighing between 18–20 gm of either sex and aged 6–8 weeks were taken. An equal number of the parasite, approx. 1 × 10^7^ cells were injected into three groups of animals; Δ*Pb*MCA-2-c1 (i), Δ*Pb*MCA-2-c4 (ii), and wild *P. berghei* ANKA (iii), containing four mice in each group. After the infection was established, 0.1ml of artemisinin was administered intraperitoneal (i.p) to each mouse at a dose of 10 mg/kg of body weight per day for the subsequent three days (day 1–3). The course of infection in drug-treated, solvent and untreated mice was monitored daily by counting the number of parasites in the Giemsa-stained smears that were obtained from the tail vein blood, and parasitaemia was determined by counting 1,000 erythrocytes [34,35].

## Sexual-stage characterization

### Phenotypic analysis of ΔPbMCA-2 parasites during sexual stages

An equal number of the parasite, approx. 1 × 10^7^ cells were injected into three groups of animals; Δ*Pb*MCA-2-c1 (i), Δ*Pb*MCA-2-c4 (ii), and wild *P. berghei* ANKA (iii), containing three mice in each group. On the third day after infection, gametocytemia (mature gametocytes per 100 RBCs) and gametocyte sex ratio were determined by Giemsa-stained blood smears [6]. For exflagellation of male gametocytes, 20μl of gametocyte-infected blood was mixed immediately with 180μl of complete ookinete culture medium (RPMI medium with 25mM HEPES, 50 mg/L hypoxanthine, 2 g/L sodium bicarbonate, 100μM xanthurenic acid, 20% human serum). The mixture was placed under a Vaseline-coated coverslip at 25°C, and 15 min later, exflagellation centres were counted over the next 10 min under a phase-contrast microscope [36]. Similarly, ookinete formation was determined using 20μl of blood collected from the tail vein of each mouse, mixed up into the 180μl ookinete culture medium and incubated at 19°C for 24 hrs. The growth of cultured ookinete was examined via Giemsa stained blood smears under a light microscope [23,37].

### Analysis of sporogonic development of transgenic parasite line

Four to six weeks old BALB/c mice were administered with phenylhydrazine solution by intraperitoneal (i.p), two days prior to injection of Δ*Pb*MCA-2 and *P. berghei* ANKA parasites. Pre-starved *An. stephensi* mosquitoes were fed at 1–2% gametocytes in infected mice incubated at 20 ± 1°C and 75 ± 5% relative humidity. On 14th day, for each mouse, midguts of ∼30 mosquitoes were dissected and stained with 0.5% mercurochrome (Sigma-Aldrich) [36]. The no. oocysts were counted to determine the intensity of infection (number of oocysts per positive midgut). Similarly, on day 20, the salivary glands of 10–20 mosquitoes were dissected, and sporozoites loads were counted by microscopic analysis [22,36]. Quantitatively, real-time PCR was performed using Forward (TAATGCCAACAGTGCTGTAA) and Reverse (CAGAGCCAGGCTTTATTCTA) RT-CSP primers. Briefly, total RNA was isolated from the oocysts and sporozoites using Trizol (Invitrogen, California) method. First-strand cDNA was synthesized using Verso cDNA synthesis Kit (Thermo Scientific, U.S). Illumina Eco Real-Time PCR machine was used to assess the gene expression by SYBR green qPCR master mix (Thermo Scientific, US). PCR parameters were used as described earlier [25]. The mosquitoes18S-rRNA gene was used as a normalizing control. Quantitative analysis was done by the measurement of threshold cycle (CT) values during the exponential phase of amplification.

## Chemical synthesis of MCA-2 inhibitory molecules

C-532 and C-533 Benzoxazole is one of the heterocyclic pharmacophore that has been known for its wide range of biological activities such as anti-cancer [38,39], anti-viral [40], anti-bacterial, [41] antioxidant, [42] and anti-inflammatory [43]. Antimalarial activity of benzoxazole derivatives is not so much explored, except the report by Tipparaju et al [43] and Ongarora et al [44], where the antimalarial activity of 2, 4- and 2, 5-disubstituted benzoxazole derivative was reported. In order to explore the antimalarial activity of benzoxazole pharmacophore, we designed two compounds based on the rational drug design approach, and the synthesis of 2, 5-disubstituted benzoxazole derivatives are summarized in Supplementary information 1). The commercially available compounds 2-amino-4-nitrophenol 6.5 µmol), cyclohexyl acetic acid, 7.2 µmol) and polyphosphoric acid (PPA), was heated at 150 °C for 5 h. After completion of the reaction, the mixture was poured into ice and neutralized by adding NaOH solution. The organic compound was extracted using ethyl acetate, and later crude organic layer was dried over Na_2_SO_4_. The crude products were purified by column chromatography which was subjected to Pd/C-H2 reduction. The amino compound thus obtained was reacted with 2-(cyclohexylmethyl) benzo[d]oxazol-5-amine (1.30 mmol), and desired aldehyde (1.30 mmol) were taken and mix with the dry ethanol followed by continuous stirring up to 5-6 h at 60 °C. After completion of the reaction, NaBH_4_ (3.90 mmol) was added at 0°C and then continued stirring for 1 h. Ethanol was removed by using rotavap, and ice-cold water was added to the reaction mixture. The desired product was extracted using ethyl-acetate solvent and finally concentrated using rotavap. The crude product obtained was purified by using column chromatography and characterized by various analytical techniques. Detailed synthesis and isolation protocols were summarized in Supplementary information 1.

## Recombinant and native *Pf*MCA-2 inhibition assay

Recombinant *Pf*MCA-2 (catalytic domain) was expressed in *E. coli* BL21DE3 and purified by Ni-NTA affinity chromatography (Qiagen, USA) as described earlier [25]. The enzymatic activity of *Pf*MCA-2 was checked by measuring the cleavage of the fluorogenic substrates Z-GRR-AMC as reported earlier [25]. Equal amounts of recombinant *Pf*MCA-2 was incubated with different concentrations of C-532 to C-533 in 50mM phosphate buffer (pH 7.4) supplemented with 5mM DTT for 10–15 min at 28^0^C. The inhibitory effect of potent compounds was also checked on the other parasite proteases like Falcipain-2 & 3 (FP-2 & FP-3), Vivapain-2 (VP-2), Plasmepsin-II (PM-II) and Metacaspase-3 (MCA-3) (Recombinant clone are available in our lab for all the enzymes used in the enzymatic assay) [1,19]. All reactions were run in duplicates, and data were analyzed using Prism and Sigma plot software. The inhibitory constant (Ki) of most effective compounds was calculated using the Michaelis–Menten equation based on nonlinear regression in Prism GraphPad software. For measuring the activity of native MCAs, we have taken the schizont-rich *P. falciparum* culture and gametocyte-rich *P.berghei* parasite. The parasite lysates were made in RIPA buffer and treated with 5µM of both C-532 and C-533. After incubation with both the inhibitors for 15 min, we have measured the activity in the presence of z-GRR.AMC substrate.

## *Plasmodium falciparum* growth inhibition assay

*P. falciparum* 3D7 (asexual culture), RKL-9 (sexual culture) and C580Y strains were cultured at 37^0^C and 5% CO_2_ in RPMI-1640 media containing 10% AB+ serum or 10% Albumax-II (Invitrogen) and 5% sodium bicarbonate, supplemented with 50 mg/l gentamycin and 50 mg/l hypoxanthine at 4-5% hematocrit in RBC [25,45,46]. Synchronization of the parasites in culture was achieved by sorbitol treatment [45], and gametocytes were obtained as described previously [47]. For the dose-dependent study of the active compounds, a semi-logarithmic serial dilution (containing ten different concentrations) was prepared at 150μM top concentration for test compounds. Parasite culture was added to a final concentration of 1% parasitaemia and 0.5% hematocrit. Plates were incubated for 36 hrs for asexual stages and 72 hrs for sexual stages at 37°C incubator with 5% CO_2_ and 95% humidity [25,48]. Blood smears of parasites were prepared after 36 hrs for asexual and 74 hrs for sexual. Quantitatively, no. of parasites in each group of animals was calculated by SYBR Green–I staining as described earlier. Statistical analysis was done by using a two-tail T-test including IC_50_ determination, and graphical output was performed in GraphPad Prism® 5 using nonlinear regression variable slope curve fitting.

## Cytotoxicity assay

Cytotoxicity of compounds was evaluated in hepatocellular carcinoma (HepG2) cells with the MTT [3-(4, 5-dimethylthiazol-2-yl)-2, 5-diphenyltetrazolium bromide] (SRL, India) assay. HepG2 cells were seeded into 96-wells plate at a density of 2 × 10^4^ cells per well and were incubated for 48 h with different concentrations (0.01mM to 10mM) of C-532 and C-533 with their respective control. After 48 h, a solution of 0.5 mg/ml MTT was added from the 5 mg/ml stock followed by incubation for 2–3 h. The resulting formazan crystals were resuspended in 50 μl of DMSO/isopropanol and further incubated for 10–15 min. Absorbance at λ_590_ nm of the cells, with medium alone (control) or different concentrations of compounds, was measured by ELISA plate reader (Techan, Switzerland). Toxicity was determined as percent viable cells, which was calculated according to the following formula: (abs_sample_− abs_blank_)/(abs_control_− abs_blank_) × 100.

## *In vivo* effect of C-532 and C-533 on sexual development of parasite

Twelve BALBc mice weighing between 18–20 gm of either sex and aged 6–8 weeks were kept in the Animal House of the National Institute of Immunology, New Delhi. For gametocytes induction, 100 µl of 6 mg/ml of phenyl-hydrazine was injected into animals. Two-day post-treatment, 100-150 µl of frozen stock of the *P. berghei* ANKA parasites were inoculated intra-peritoneal (i.p) into the mice's [23]. After 5–6 days of infection (∼5-8% parasitaemia), all mice were sacrificed, and infected blood from each mouse was collected separately in the heparinized tube. An equal number of the parasite, approx. 1 × 10^7^ cells were further injected into four groups of animals containing four mice in each group. After the infection was established, 0.1 ml of respective molecules, C-532 and C-533 was administered intraperitoneal (i.p) to each mouse at a dose of 10 mg/kg of body weight per day for subsequent five days (day 1–5). The course of infection was monitored as described earlier [25,34].

## Results

### PbMCA-2 knockout confirmation

The *Pb*MCA-2 was knocked out by homologous recombination (Supplementary Figure.1A). *Pb*MCA-2 knockout-line (Δ*Pb*MCA-2) were selected by pyrimethamine and cloned by limiting dilution method. The deletion of the *Pb*MCA-2 gene was confirmed by the integration site-specific PCR with the expected size of amplicons size of ∼0.9 kb was observed in the selected MCA-2 knockout clones 1 and 4 as compared to wild-type amplicons (∼0.7kb) (Supplementary Figure.1B). Moreover, the absence of expected band size of ∼190 KDa in Δ*Pb*MCA-2 parasite lysate and no FITC positive signal in Δ*Pb*MCA-2 parasites after probing with anti-*Pf*MCA-2 antibody confirmed the MCA-2 deletion (Supplementary Figure.1C and 1D). Southern blotting further confirmed the deletion of MCA-2 (data not shown).

### ΔPbMCA-2 line displayed features of oxidative stress

Compared to wild-type, the level of ROS was significantly higher in Δ*Pb*MCA-2 line within 2 hrs (*P* = 0.005), and the level further increased over 8 hrs (Supplementary Figure. 2A). Furthermore, the viability of parasites was measured using AlamarBlue dye. The fluorescent signal from the wild-type parasites was constantly increased, whereas the signal was declined in Δ*Pb*MCA-2 line at 8 hrs (Supplementary Figure.2B). The decline in fluorescence signal indicated that the majority of parasites lost their viability within 8 hrs (*P* = 0.005). A deletion of *Pb*MCA-2 significantly affected the calcium level, mitochondrial potential and ATP level inside the cell. Notably, the intracellular [Ca^2+^] level was optimally measured in wild type parasites at 46 ± 3nM. However, Δ*Pb*MCA-2 line showed a time-dependent increase in cytosolic Ca^2+^, approx. 158 ± 3.2nM was observed after 8 hrs of incubation (*P* = 0.005) (Supplementary Figure. 2C). The changes in the mitochondrial membrane potential (ΔΨm) were determined using mitosensor dye JC-1. There is a significant decline in the ratio of red/green fluorescence intensity (*P* = 0.009) in Δ*Pb*MCA-2 parasites suggesting the occurrence of mitochondrial depolarization (Supplementary Figure.2D). Further, a fall in the mitochondrial membrane potential of Δ*Pb*MCA-2 parasites was indicated by increased green fluorescence intensity and loss of red signal (Supplementary Figure. 2E). We also measured intracellular ATP content in Δ*Pb*MCA-2 line. Interestingly, after 8 hrs, there was a drastic decrease in ATP level (53.6nmol/106cells) in Δ*Pb*MCA-2 parasites compared to wild-type 124nmol/106 cells (Supplementary Figure. 2F). These results indicated that the generation of ROS followed by depolarization of mitochondrial potential (ΔΨm) inhibited the cellular ATP generation.

### ΔPbMCA-2 parasite line undergoes programmed cell death

To study cell death, parasites with 1-2% parasitaemia were taken, and the percentage of cells undergoing apoptosis was determined by flow cytometry after staining with annexin V-FITC and propidium iodide (PI). In Δ*Pb*MCA-2 line, approx. 61% of cells undergo apoptosis-like cell death than wild-type (∼18%) (Supplementary figure 3A and 3B). Notably, ∼60% of Δ*Pb*MCA-2 parasites were positive for TUNEL staining in the presence of terminal deoxynucleotidyl transferase (TdT), and a much smaller proportion was stained in the absence of TdT (Supplementary Fig. 3C and 3D). Microscopic analysis revealed DNA fragmentation in 8-nucleated schizonts in the Δ*Pb*MCA-2 line (Supplementary Figure.3E). Flow cytometry analysis also revealed that the percentages of TUNEL-positive cells were increased in Δ*Pb*MCA-2 line compared to wild-type (13%) (Supplementary Figure 3C and 3D). Our findings indicated that deletion of *Pb*MCA-2 induces apoptosis-like cell death in *Plasmodium*.

### PbMCA-2 is important for the sexual development of parasite

**Figure 1.**
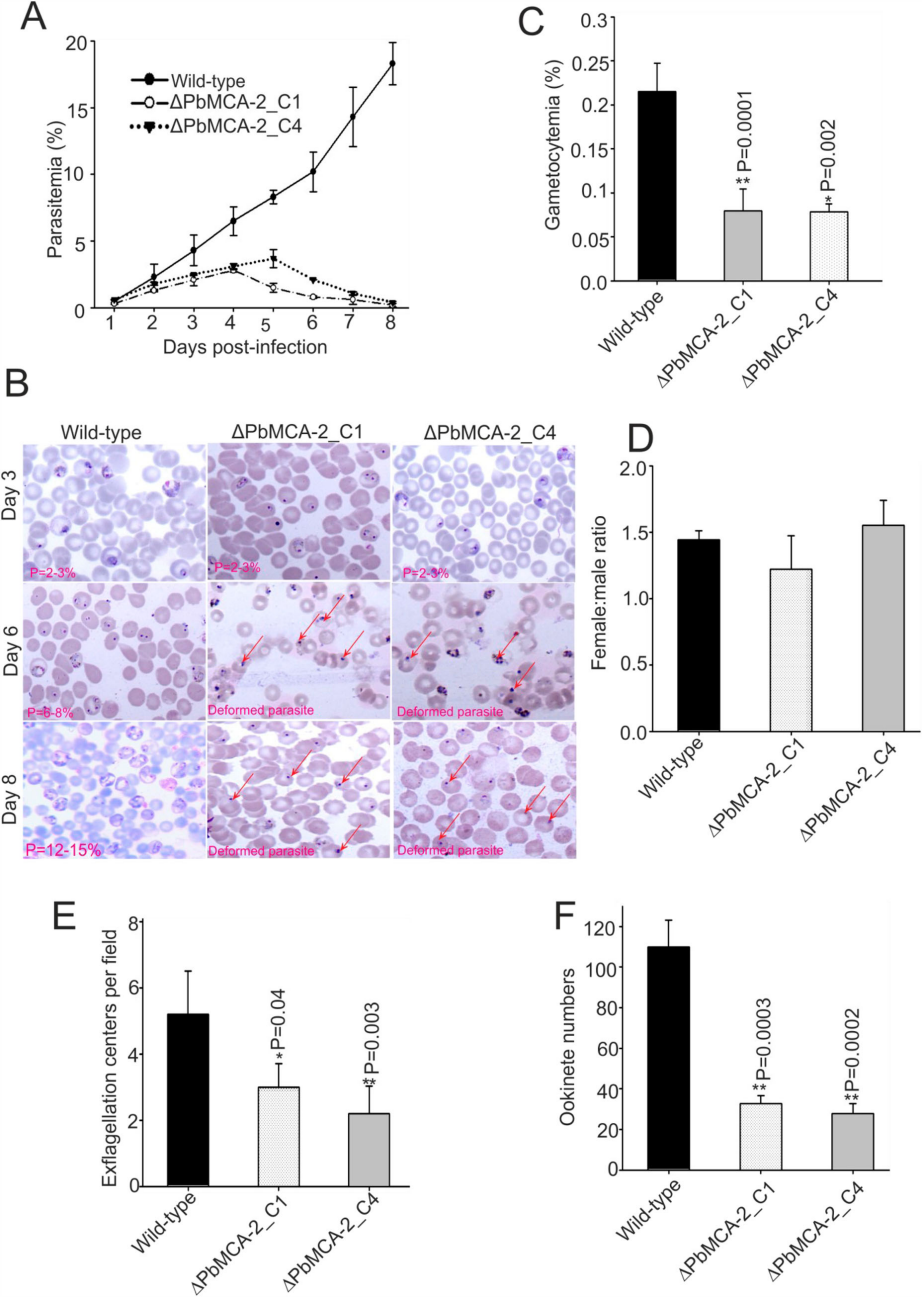
**Functional analysis of MCA-2 during parasite (asexual and sexual) development**. Mice were infected with *P. berghei wild* or Δ*Pb*MCA-2 parasite, and parasitaemia was monitored for 11 days **(A)**. Giemsa stained bright-field microscope images were showing the predominant phenotype asexual stages of the parasite during the course of infection in the wild and ΔPbMCA-2 parasite line **(B)**. Gametocytemia in mice infected with wild-type and Δ*Pb*MCA-2 parasites. **P < 0.001 (Student t-test) **(C)**. Female: male gametocyte ratios of wild and Δ*Pb*MCA-2 parasites **(D)** Exflagellation of male gametocytes in the wild-type and Δ*Pb*MCA-2 parasites; **P < 0.04 (Student’s t-test) **(E)**. Ookinete numbers in wild and Δ*Pb*MCA-2 parasites **(F)**. **P < 0.002 (Student’s t-test). The error bars in B-E indicate mean ± SD (n = 10).

Phenotypic analysis of the wild-type and Δ*Pb*MCA-2 parasite was performed in both asexual and sexual stages. Consistent with our previous study, inhibition of *Pf*MCA-2 by its inhibitor caused abnormal parasite growth *in-vitro* [23]. The deletion of the *Pb*MCA-2 gene did not show a noticeable difference in growth at low parasitaemia, but once the parasitaemia reached >3-5%, the parasites attained deformed morphology and were probably unable to grow further in the parasite cycle (Figure 1A and 1B). These findings suggested that *Pb*MCA-2 could be important for the normal parasite growth and maintenance of high parasitaemia. However, compared to the wild type, the Δ*Pb*MCA-2 line displayed a significant reduction in gametocytes formation (*P* = 0.0001; Figure 1C). Gametocytemia reduced to 2.7 folds in the Δ*Pb*MCA-2 parasites as compared to wild type. Furthermore, there was no significant difference in the sex ratio between the wild-type and Δ*Pb*MCA-2 parasites (Figure 1D).

### Deletion of MCA-2 impairs the parasite cycle during mosquito stage

**Figure 2.**
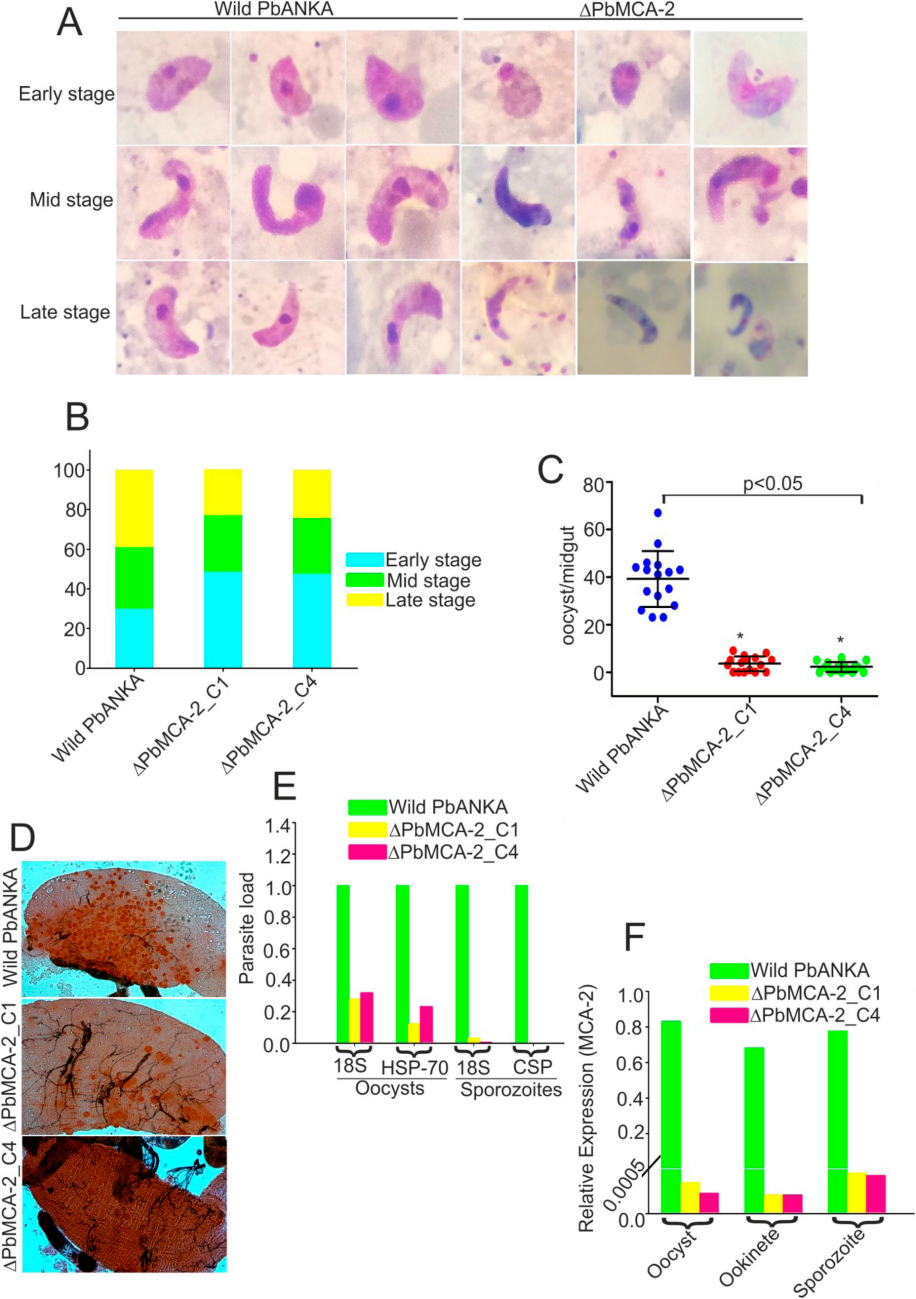
**Functional analysis of MCA-2 during sporogonic development.** Giemsa stained images of ookinete morphology in wild-type and Δ*Pb*MCA-2 parasites **(A)**.Graphical representation of the developmental pattern of the ookinete in wild-type and Δ*Pb*MCA-2 parasites **(B)**.Oocysts number per midgut in mosquitoes fed on WT and Δ*Pb*MCA-2 parasites. **P < 0.01 (student t-test) **(C)**. Mercurochrome stained midgut images showing oocysts numbers in the mosquito fed on WT and Δ*Pb*MCA-2 parasites **(D)**. Bar graph showing the quantitative difference of the oocysts and sporozoites load in the mosquito upon infection with WT and Δ*Pb*MCA-2 parasites **(E)**. A bar graph showing the mRNA expression of MCA-2 in the different stages (oocysts, ookinete and sporozoites) of the parasite **(F)** Data in the functional analysis of *Pb*MCA-2 are representative of three independent experiments.

To determine the role of *Pb*MCA-2 in subsequent parasite development in the mosquito host, we measured exflagellation and ookinete conversion from equal numbers of gametocytes. Notably, there was a ∼50–60% reduction in exflagellation centres/field (*P* = 0.003; Figure 1E) in Δ*Pb*MCA-2 line compared to the wild-type line. Moreover, a significant reduction (>70%) in ookinete number was observed in the Δ*Pb*MCA-2 parasite line (*P* = 0.003; Figure 1F) compared to wild-type. Morphological deformities were observed in the ookinete, and the propagation of early to late ookinete was also severely affected in Δ*Pb*MCA-2 line (Figure 2A and 2B). The majority of the ookinete was arrested in the early stages compared to the wild type (Figure 2B). The Δ*Pb*MCA-2 line displayed a significant reduction in oocysts load (>90%) in the midguts as compared to wild-type (Figure 2C and 2D). Similarly, a drastic decrease in sporozoites number was also observed in the Δ*Pb*MCA-2 parasite line (Figure 2E). We further assessed the relative expression level of the MCA-2 in oocysts, ookinete and sporozoites stages. It was observed that the MCA-2 was uniformly expressed in oocysts, ookinete and sporozoites stages (Figure 2F). In contrast, no expression was observed in all three stages in the case of MCA-2 knock out parasites (Figure 2F).

### Deletion of MCA-2 causes parasite drug susceptibility

**Figure 3.**
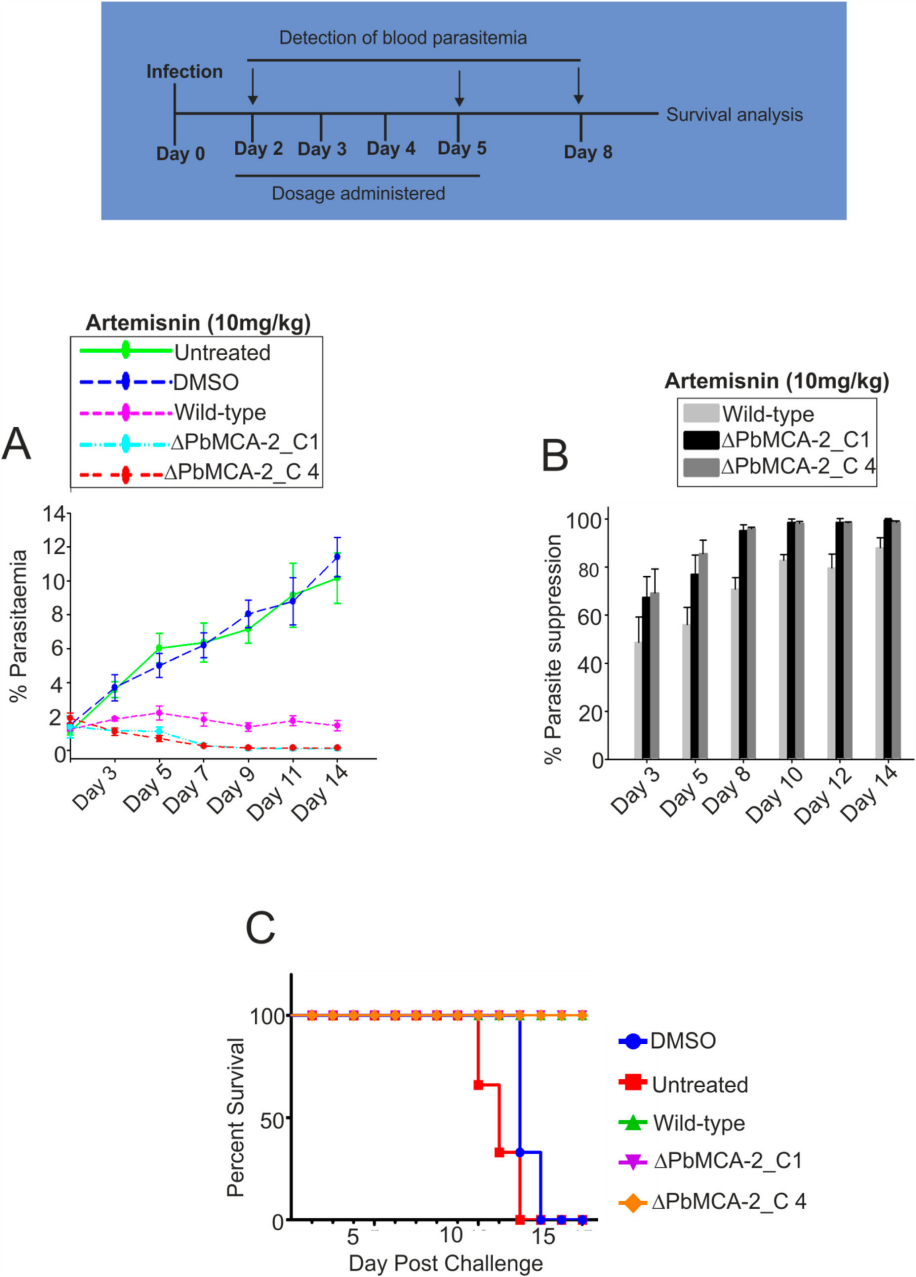
**Effect of Artemisinin drug in the Δ*Pb*MCA-2 parasites**. Mice in each experimental group were treated with the compound Artemisinin (10 mg/kg). The per cent parasitaemia from days 3–14 shown in the figures, on day 12, the mean parasitaemia was determined to be <10% (untreated), whereas in the Artemisinin group the mean parasitaemia was >3% **(A)**. The bar graphs represented the percentage of parasite suppression in treated groups over a period of time **(B)**. A graph represented the survival rate of animals after drug treatment in the Δ*Pb*MCA-2 and wild-type parasites **(C)**.

We assessed the effect of current antimalarial drug, artemisinin in the wild and Δ*Pb*MCA-2 line using murine parasite, *P. berghei*. The parasitaemia was drastically reduced just after the first dose of artemisinin in Δ*Pb*MCA-2 treated parasite line compared to the control DMSO. However, in the case of the wild-type parasite line, parasitaemia was reduced after five days of treatment (Figure 3A and 3B) (Supplementary Table. 2). A decline in parasitaemia enhances the survival rate of a treated animal as 100% survival was observed in Δ*Pb*MCA-2 parasite-infected mice compared to control DMSO (Figure 3C).

### C-532 and C-533 inhibits recombinant PfMCA-2

**Figure 4.**
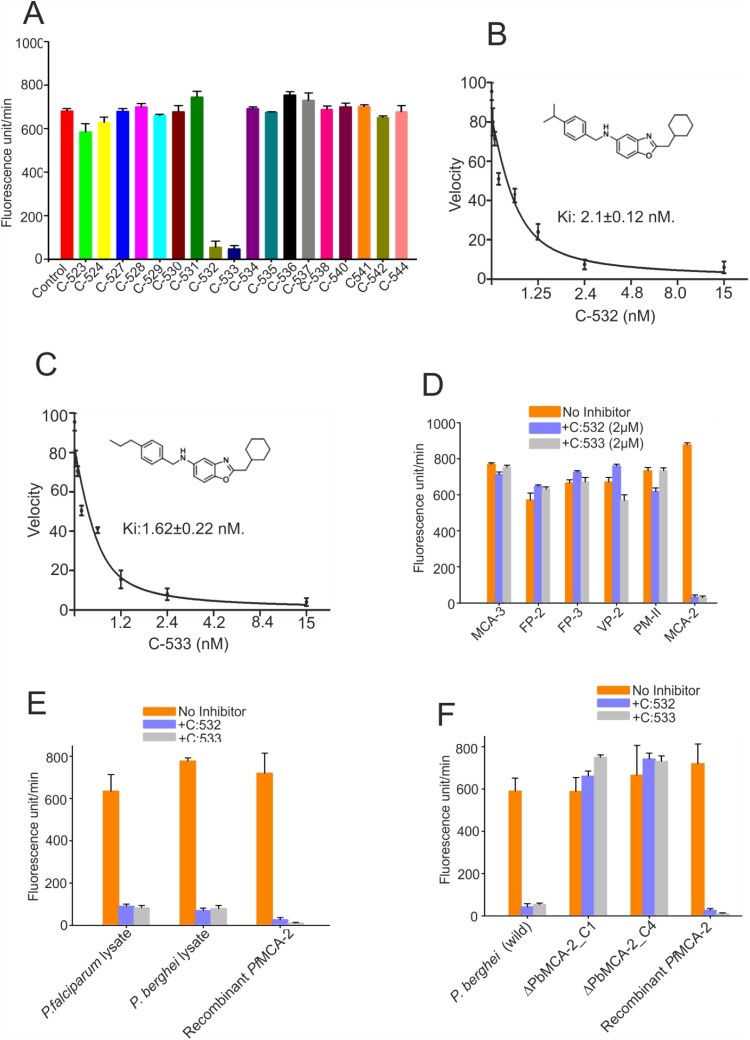
**Enzymatic activity of *Pf*MCA-2 in the presence of different compounds.** Different compounds were tested against MCA-2. Eighteen compounds were checked at 2 µM concentration **(A)**. Kinetic study of *Pf*MCA-2 using different concentrations of the effective compounds, C-532 and C-533, and Ki values were calculated based on nonlinear Michaelis–Menten regression **(B, C)**. C-532 and C-533 had no effect on the activities of well-characterized malaria proteases, MCA-3, FP-2, FP-3, VP-3 and PM-II **(D)**. Native MCAs enzymatic activity using z-GRR-AMC substrate and inhibitory molecules, C-532 and C-533 in *P. falciparum* and *P. berghei* lysates **(E)** Native MCAs enzymatic activity using z-GRR-AMC substrate and inhibitory molecules, C-532 and C-533 in *P. berghei* wild and MCA-2 knockout parasites **(F)**

We assessed the inhibition of *Pf*MCA-2 by 18 test compounds (designed based on a modification in the skeleton of an earlier reported compound, SS-5) [20]. Among the 18 compounds, only C-532 and C-533 showed ∼90% inhibition of the *Pf*MCA-2 at 2 µM, whereas the rest of the compounds had no effect on the enzyme activity (Figure 4A). However, except C-532 and C-533, other compounds were not inhibitory at 50µM concentration (Supplementary figure. 4). A kinetic study of the enzyme with the most effective compounds, C-532 and C-533 was performed based on nonlinear Michaelis–Menten regression. The calculated Ki of C-532 and C-533 was 2.1 ± 0.12nM and 1.62 ± 0.22nM, respectively (Figure 4B and 4C). To consider the specificity of protease inhibition, we assessed the activities of other selected proteases. The activities of selected *P. falciparum* proteases (Metacaspase-3, falcipain-2, falcipain-3, vivapain-2 and plasmepsin-II) were not inhibited in the presence of C-532 and C-533 (Figure 4D). Therefore, among the test compounds, C-532 and C-533 were effective inhibitors of *Pf*MCA-2. We have further assessed the *in-vivo* activity of MCA-2 using its specific substrate, z-GRR-AMC. Using the *P. falciparum* and *P. berghei* lysates, the increased fluorescence units in terms of z-GRR-AMC cleavage were observed, which indicated the activity of native MCAs. Notably, few fluorescence units were observed in the presence of C-532 and C-533 in schizont-rich *P. falciparum* and gametocytes-rich *P.berghei* lysates possibly indicated the activity of minimally expressed MCA-1 or MCA-3 (maximal expression were reported in ring and trophozoites stages) [16,19] in respective stages of the parasite (Figure 4E). However, fluorescence activity of MCAs was detected in the Δ*Pb*MCA-2 parasite lysate in the presence of both the inhibitory molecules, C-532/C-533, indicating that C-532 & C-533 are not inhibiting MCA-3 (Figure 4F).

### C-532 and C-533 block P. falciparum asexual and sexual growth in vitro

**Figure 5.**
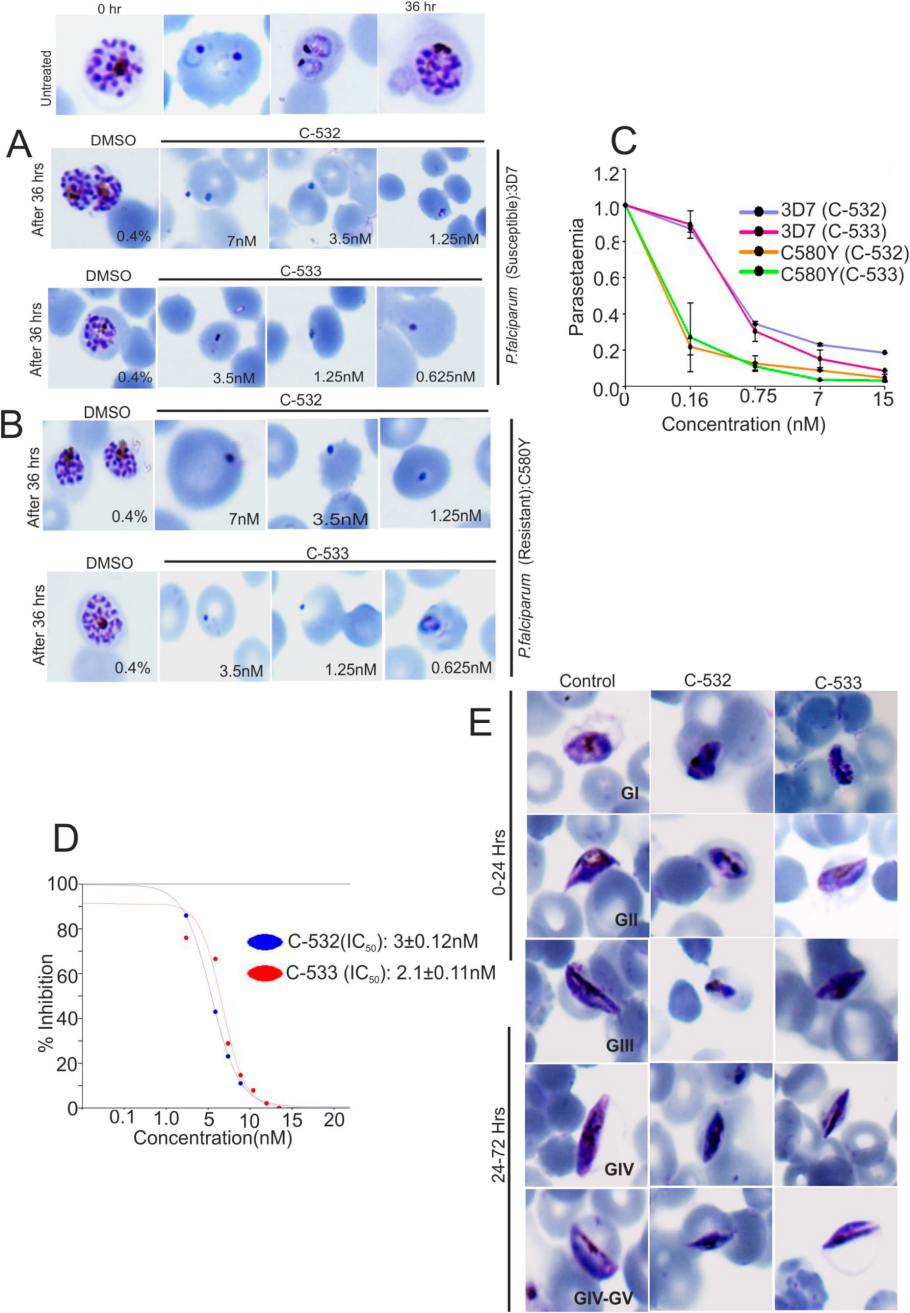
***In vitro* inhibition of *P. falciparum* growth at asexual and sexual stages in the presence of MCA-2 inhibitors, C-532 and C-533.** Giemsa stained bright-field microscope images showed the predominant parasite phenotype at different concentrations of C-532 and C-533 after 36 hrs of incubation. Treated parasites did not progress normally at their lowest concentration of 0.6nM, and morphologically distorted rings were observed **(A, B)**. Solid lines graphs represented the parasitaemia in the C-532, and C-533 treated parasites after staining with syber-green-I dye **(C, D)**. Giemsa stained bright-field microscope images were showing the predominant phenotype of the gametocytes stages of the parasite at 24 and 72 hrs after the addition of compounds C-532 and C-533 **(E)**. A graph represented the IC_50_ value of C-532 and C-533 for inhibiting 50% parasite growth *in vitro* **(F)**.

To examine a stage-specific action, C-532 and C-533 were added to the culture of *P. falciparum* (3D7 strain) at the schizonts stage. C-532 and C-533 treated schizont stage parasites were ruptured and developed into an abnormal ring. Moreover, the transition from abnormal ring to further stages was halted in the treated parasites (Figure 5A). C-532 and C-533 also inhibited the growth of the artemisinin resistant parasite, C580Y strain (Figure 5B). Quantitatively, a significant reduction in parasitaemia was observed after SYBR green-I staining of C-532 and C-533 treated parasite compared to untreated control (Figure 5C). The IC_50_ for the growth inhibition of 3D7 parasite by C-532 and C-533 was 3 ± 0.21nM and 2.1 ± 0.11nM, respectively (Figure 5D). Similarly, in sexual stages, deformed gametocytes (stage-II, III and IV) were observed after treatment with C-532 and C-533 at 24-hrs and 72-hrs (Figure 5E). Approx. >50% of gametocytes growth was abolished in the presence of C-532 and C-533.

### C-532 and C-533 inhibits sexual growth in vivo

**Figure 6.**
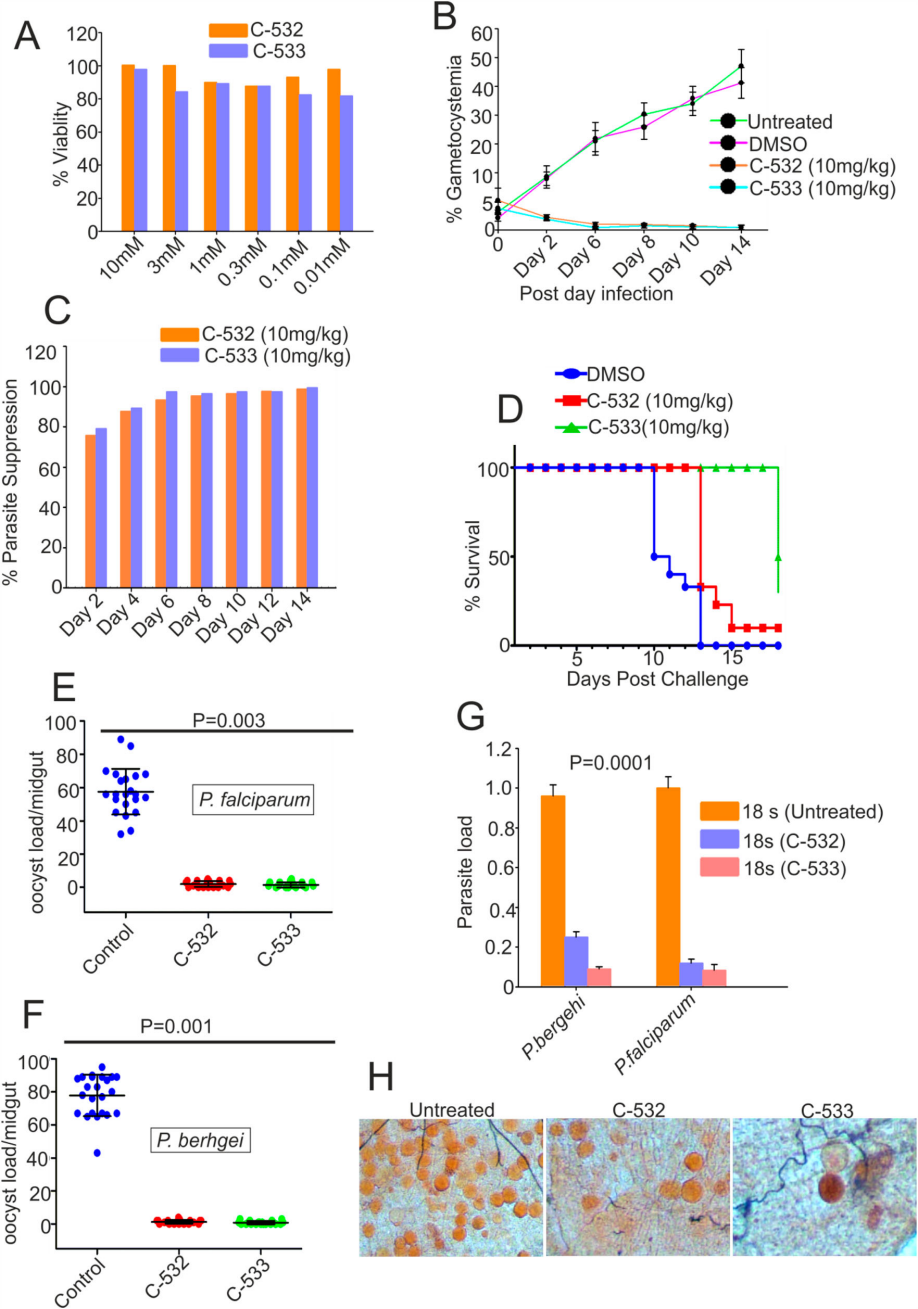
**Effect of C-532 and C-533 on sexual cycle *in-vivo* and their transmission-blocking activity.** The percentage cell viability was assessed in HepG2 cell line in the presence of C-532 and C-533 and their respective controls **(A)**. The 10mg/kg of both C-532 and C-533 was injected in *P. berghei* infected mice. The percent gametocytemia from days 0–14 is shown in the figure. On day 14, treated groups showed significantly low parasite load compared to control **(B)**. A bar graph depicted the percent parasite suppression in the treated groups **(C)**. Graph showing the survival of animals after C-532 and C-533 treatment **(D)**. The effect of C-532 and C-533 on the transmission of *Plasmodium sp*. in the malaria vector was assessed. The 10 nM concentrations were used for C-532 and C-533 in *P. falciparum* gametocyte culture and 10mg/kg in the *P. berghei* infected mice. Dots plot showing oocysts count per midgut in *An. stephensi* after feeding with *P. falciparum* mature gametocytes rich culture, and mice infected with *P. berghei* ANKA **(E and F)**. Graph showing the *P. falciparum and P. berghei* infectivity in the *An. stephensi* by measuring 18s rRNA expression using real-time PCR **(G)**. Mercurochrome stained midgut images showing oocysts numbers in the mosquito fed on wild parasites and treated with C-532 and C-533 **(H)**.

We assessed the activities of C-532 and C-533 against the murine parasite *P. berghei*. The cytotoxicity of both the compounds was evaluated in the hepatocellular carcinoma (HepG2) cell line by MTT assay. After 48 hrs incubations with up to 10 mM of C-532 and C-533, the cells had 70-80% viability, indicated a lack of marked toxicity (Figure 6A). Interestingly, after three days of treatment to *P. berghei* infected mice with C-532 and C-533, there was a significant reduction (>95%) in the gametocytemia compared to control and more than 70% parasite suppression on day 2nd was observed (Figure 6B and 6C). Parasite clearance further prolonged the mice survival by 70% in the treated groups (Figure 6D).

### C-532 and C-533 block the transmission of P. falciparum and P. berghei

To test whether C-532 and C-533 exhibit transmission-blocking activity against *Plasmodium sp*, we incubated 10nM of C-532 and C-533 into gametocytes rich culture of *P. falciparum* (*in vitro*) and 10mg/kg of both the compounds were injected in *P.berghei* infected mice (*in vivo*). Further, an infected culture fed to *An. stephensi*. The average number of oocysts per midgut was reduced from 58 to 2.0 and 1.4 in *P. falciparum* for C-532 and C-533, respectively. Similarly, in the case of *P. berghei*, oocysts load was reduced from 78 to 1.2 and 0.8, for C-532 and C-533, respectively (Figure 6E and 6F). C-532 and C-533 significantly reduced the number of oocysts of the *P. falciparum* and *P. berghei* by more than 95% (Figure 6E, 6F and 6H). A real-time PCR further showed a reduced expression of parasite 18s rRNA gene in treated groups, which confirmed that C-532 and C-533 significantly reduced the parasite burden in mosquito host (Figure 6G).

## Discussion

Interrupting the development of a parasite at multi-stages of its life cycle, primarily its transmission inside the mosquito vector, is a critical component of malaria control strategies. In this study, using systemic gene knockout, we have revealed the functions of metacaspase-2 (MCA-2) in the multi-steps of the *Plasmodium* life cycle. Based on the sequential conservation of MCA-2 among the *Plasmodium* species, we generated a knockout parasite in *P. berghei* using a gene replacement strategy. We demonstrate that *Pb*MCA-2 regulates stress-mediated cell death at the blood stage development, whereas at the exo-erythrocytic stages, its deletion led to the significant reduction in the number of gametocytes, followed by ookinete, oocysts, and sporozoites load indicating MCA-2 is important for sustaining the parasite load/infection throughout the life cycle. Additionally, MCA-2 inhibitory molecules, C-532 and C-533, inhibit the MCA-2 activity along with parasite growth *in vitro* and *in vivo* and disrupt the *Plasmodium* transmission.

To delete the endogenous *Pb*MCA-2, we generated a targeting construct comprising constitutively expressing GFP, and human dihydrofolate reductase (hDHFR) cassettes, flanked by 5’ and 3’ untranslated regions (UTRs) of *Pb*MCA-2. By limiting dilution, we successfully screened the Δ*Pb*MCA-2 clone-1 (C1) and clone 4 (C4). We examined the phenotype of Δ*Pb*MCA-2 parasite during the *Plasmodium* life cycle progression. In accordance with our earlier reports where tight inhibition of MCA-2 by its inhibitor generates stress inside the parasite and causes apoptosis-like cell death [20]. Interestingly, a complete deletion of MCA-2 also dysregulates the vital cellular machinery in terms of forming reactive oxygen species, loss of cell viability, mitochondrial depolarization and reduced ATP generation followed by cell death (Supplementary Figure 2 and 3). However, comparing the growth at the blood stage, initially, Δ*Pb*MCA-2 parasites grow normally at low parasitaemia, but once the parasitaemia reached >5%, the parasite growth ceases, and cells become sick, probably non-viable & displayed features of apoptosis-like cell death (Supplementary Figure 2 and 3; Figure 1A). Taken together, these data confirm that MCA-2 is important for the maintenance of high parasitaemia and the regulation of stress-dependent cell death, as reported earlier [20,25].

We further sought to investigate the developmental fate of Δ*Pb*MCA-2 parasites during the sexual cycle and in the mosquito vector. Interestingly, the gametocytes number was significantly reduced in Δ*Pb*MCA-2 parasites without any observable difference between the male to female sex ratio (Figure 1D). These results suggested that MCA-2 could be important for the development of gametocytes. Furthermore, *Pb*MCA-2 was uniformly expressed in the oocysts, ookinete and sporozoites. The number of ookinete, oocysts & sporozoites produced by Δ*Pb*MCA-2 infected mosquitoes were lesser in number compared to wild-type parasites, and the sporozoites were lately causing infection to naïve mice (*P* = 0.003; Figures 1 and 2) (Supplementary Table.3). Delayed infection possibly due to defects in sporozoites formation as a result of MCA-2 disruption or that the sporozoites density failed to reach a critical threshold at any given point for the timely infection in mice. To support this argument, approximately100,000 sporozoites were needed to cause a blood-stage infection in mice infected with *Pb*TRAP knockouts parasite [49,50]. A deletion of MCA-2 adversely affected the mosquito stage development of parasites as shown by defective ookinete, a small number of oocysts and a lower number of sporozoites that caused delayed infection in mice. These findings indicate an important role of MCA-2 in sporogonic development. Moreover, the Δ*Pb*MCA-2 parasites were more sensitive to drug treatment as only a single dose of artemisinin cleared >60% parasite load and prolonged the survival of mice (Figure 6). This further suggested that MCA-2 could be important for sustaining normal parasite growth under drug pressure, as evident for MCA-1 defective parasite undergoes apoptosis-like cell death upon chloroquine treatment [17].

The results so far suggested that MCA-2 is critical for the propagation of the *Plasmodium* cycle in the malaria vector. Moreover, its uniform expression from gametocytes to sporozoites indicates that its inhibitory molecule might interrupt parasite transmission at multiple steps. These observations led us to screen potent MCA-2 inhibitors, preferentially attributed with multi-stage activity. We identified two non-cytotoxic multi-stage active compounds, C-532 and C-533, which remarkably inhibits the recombinant MCA-2 activity *in vitro* (Figure 4A). C-532 and C-533 also specifically block the action of native MCA-2 in schizont-rich *P. falciparum* and gametocytes-rich *P. berghei* lysate, where it was maximally expressed compared to MCA-1 or MCA-3, which are known to be expressed in early rings and trophozoites stages [16,19]. In accordance with our previous findings [20], optimum MCA-2 level is proven to be important for the survival of parasites as its inhibition leads to the generation of oxidative stress responses by either inducing apoptosis or preventing DNA damage recovery responses [25]. Further, C-532 and C-533 abolished the growth of both asexual blood stages and caused deformed sick gametocytes parasites (Figure 5A and 5E). The mechanism of action of C-532 and C-533 in blocking erythrocytic, sexual growth and transmission via MCA-2 inhibition could be correlated as with MCA-2 deletion, Δ*Pb*MCA-2 parasite showed 2.7 folds reduced gametocytemia and 50-60% reduced rate of exflagellation. Therefore, we predicted that C-532 and C-533 possibly hindered the parasite's ability to form mature V stage gametocytes or block exflagellation, thereby impairing the *Plasmodium* transmission. These observations suggested that MCA-2 is important for the *Plasmodium* transmission cycle in the mosquito host*.* Notably, C-532 and C-533 also inhibited the growth of the artemisinin-resistant parasite *in vitro;* therefore, they could be further assessed in combination with artemisinin for their implementation in artemisinin-combination therapy (ACT).

**Figure 7.**
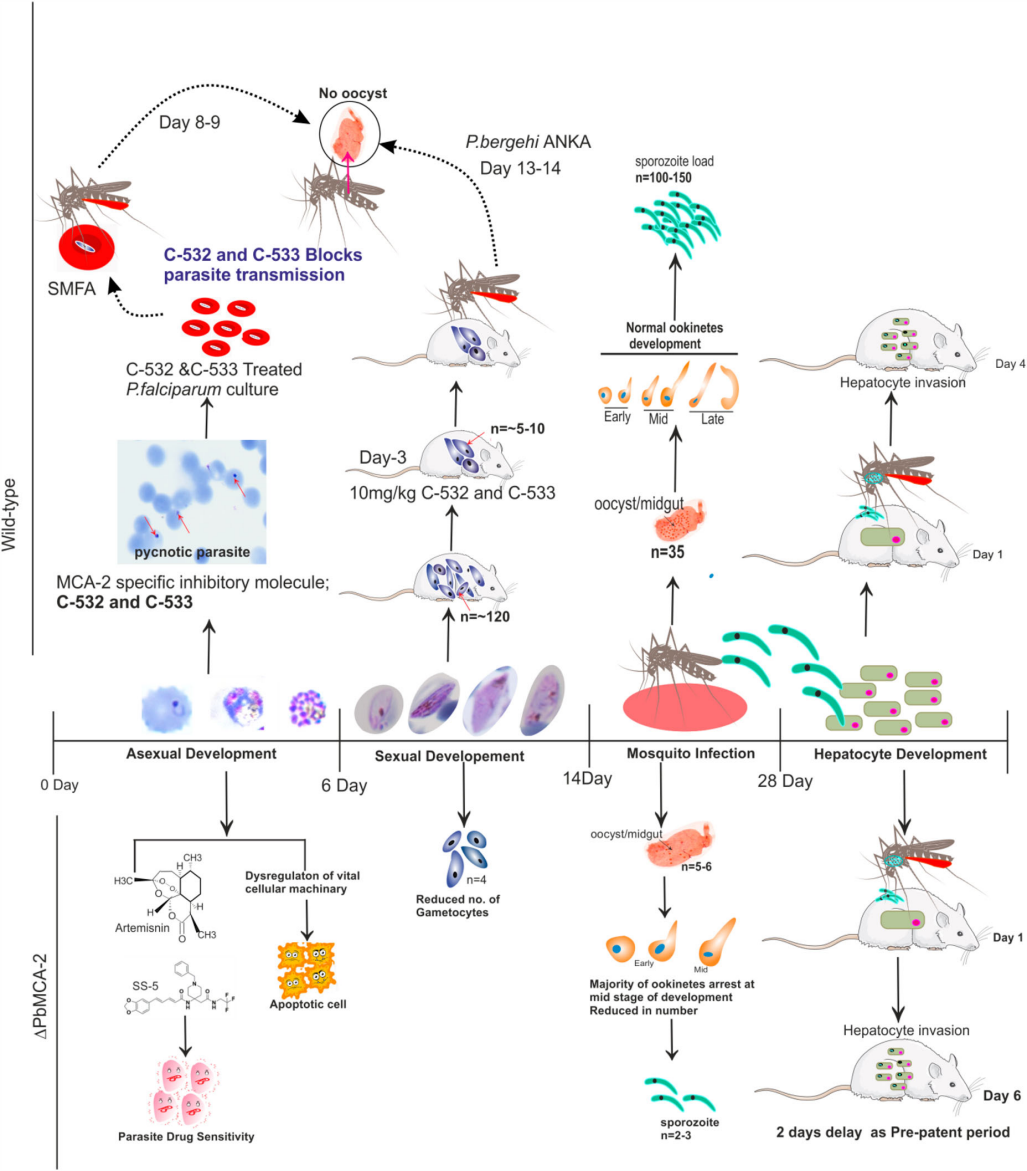
A summarized graphical depiction of MCA-2 function in the Δ*Pb*MCA-2 parasites and action of potential MCA-2 specific inhibitory molecules, C-532 and C-533.

Deletion of MCA-2 caused the growth deterioration at high parasitaemia and stress-dependent cell death in the mixed parasite population at the erythrocytic stage. Notably, there is no complete deterioration/inhibition of growth in all parasite cells at every stage; few parasite cells are retained their average growth and able to progress the parasite cycle from blood to exo-erythrocytic stages in Δ*Pb*MCA-2 parasite line. Overall, this study reveals that the *Pf*MCA-2 appears to be an important enzyme for regulating the *Plasmodium* life cycle in human and mosquito vector hosts. As indicated by the results of this study, *Pf*MCA-2 is important for the propagation of the sexual and sporogonic cycle, specifically gametocytes formation and development of a sufficient number of infectious ookinete, oocysts and sporozoites inside the mosquito host (Figure 7). The MCA-2 inhibitory molecules, C-532 and C-533, could be further implemented in blocking the transmission of the malaria parasite.

## Supplementary Material

Supplemental Material
