# Supplementary material for: Dissecting The role of Plasmodium metacaspase-2 in malaria gametogenesis and sporogony
Source: Emerg Microbes Infect. 2022 Mar 30;11(1):938–55. doi: 10.1080/22221751.2022.2052357 (PMC8973346; doi:10.1080/22221751.2022.2052357)
Supplement: Supplemental Material [file TEMI_A_2052357_SM7406.doc]

**Dissecting the role of *Plasmodium* Metacaspase-2 in malaria gametogenesis and sporogony**

Vandana Kumari^1^, Kona Madhavinadha Prasad^1^, Inderjeet Kalia^2^, Gagandeep Sindhu^3^, Rajnikant Dixit^1^ , Diwan S Rawat^3^, O. P Singh^1^, Agam P Singh^2^, Kailash C Pandey*^1,4^

1. ICMR-National Institute of Malaria Research, New Delhi
2. National Institute of Immunology, New Delhi
3. Dept. of Chemistry, University of Delhi, New Delhi
4. Academy of Scientific and Innovative Research (AcSIR), UP

**Highlights**

- Deletion of Metacaspase-2 (MCA-2) caused an unregulated stress-mediated apoptosis-like cell death and reduced the formation of gametocytes.
- A significant reduction in oocysts, ookinete and sporozoites load, and delay in hepatocytes invasion in the Δ*Pb*MCA-2 parasites.
- MCA-2 inhibitory molecule, C-532 and C-533, inhibits *Pf*MCA-2 activity along with the parasite asexual and sexual growth *in vitro* and *in vivo*.
- C-532 and C-533 impair *Plasmodium* transmission in *An. stephensi*, possibly by inhibiting MCA-2.

**Summary**

The family of apicomplexan specific proteins contains caspases–like proteins called "metacaspases". These enzymes are present in the malaria parasite but absent in human; therefore, these can be explored as potential drug targets. We deleted the MCA-2 gene from *Plasmodium* *berghei* genome using a gene knockout strategy to decipher its precise function. This study has identified that MCA-2 plays an important role in parasite transmission since it is critical for the formation of gametocytes and for maintaining an appropriate number of infectious sporozoites required for sporogony. It is noticeable that a significant reduction in gametocyte, oocysts, ookinete and sporozoites load along with a delay in hepatocytes invasion were observed in the MCA-2 knockout parasite. Furthermore, a study found the two MCA-2 inhibitory molecules known as C-532 and C-533, which remarkably inhibited the MCA-2 activity, abolished the *in vitro* parasite growth, and also impaired the transmission cycle of *P. falciparum* and *P. berghei* in *An. stephensi*. Our findings indicate that the deletion of MCA-2 hampers the *Plasmodium* development during erythrocytic and exo-erythrocytic stages, and its inhibition by C-532 and C-533 critically affects the malaria transmission biology.

**Keywords**: Malaria, Metacaspase, Specific inhibitor, *Plasmodium* transmission, Sporogony, *An. stephensi*, Gametogenesis, Ookinete


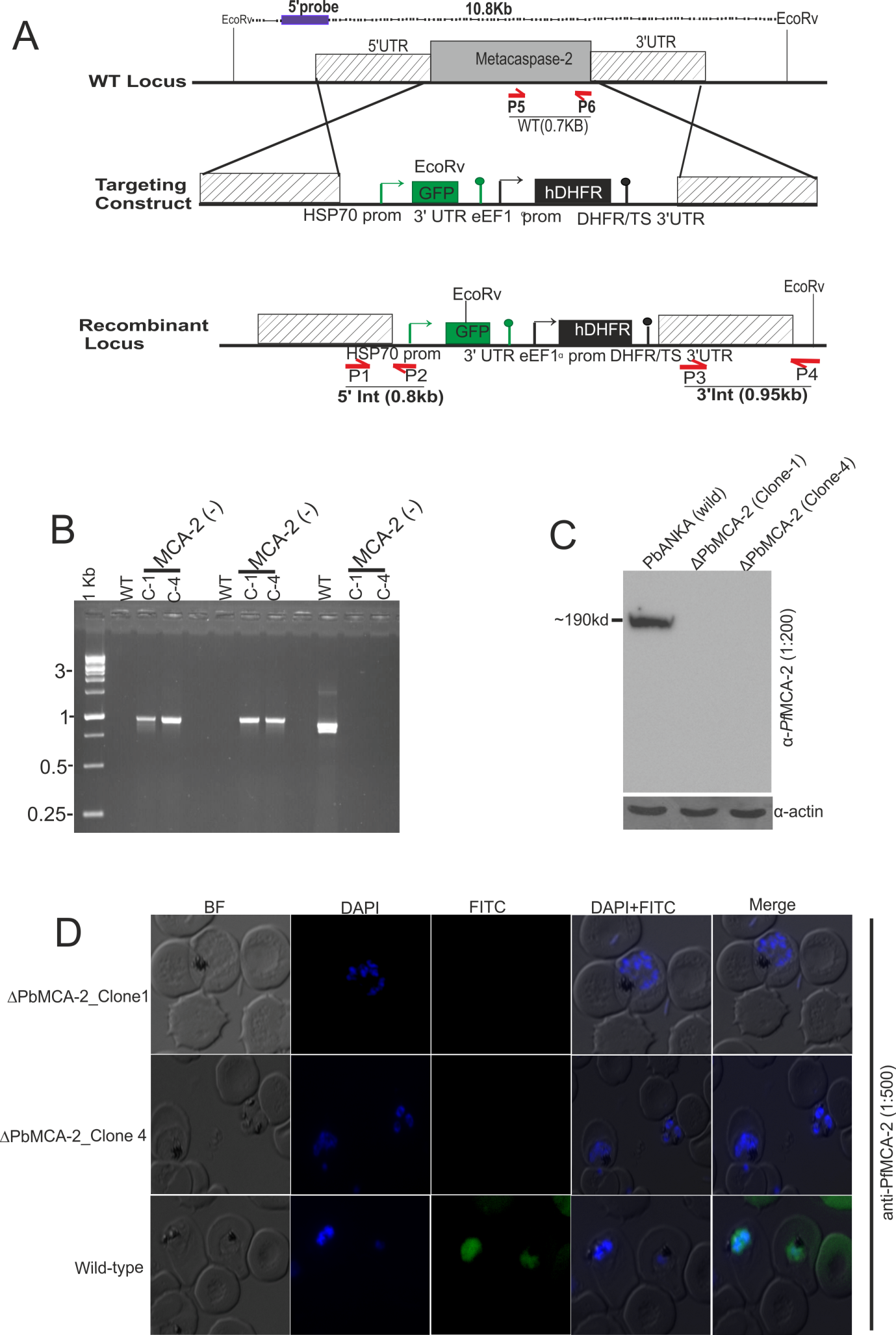


**Supplementary Figure 1: Knockout of *Pb*MCA-2 gene from *P. berghei* parasite**. A schematic shows the wild type *Pb*MCA-2 locus, transfection construct, and the recombined locus. Using a double-crossover strategy, *Pb*MCA-2 locus was replaced with the Hdhfr-expressing cassette **(A).** The gene deletion (knockout) was confirmed by diagnostic PCR analysis. The integration (Int) at 5' and 3' UTR ends were confirmed by primer combinations that amplified the recombinant locus, P3 + P4 for 3' end (~0.9 kb) in case Δ*Pb*MCA-2 (c1 and c4) and absence of a wild type (WT) locus was confirmed with primers P5 + P6 (~0.7kb) **(B).** Western blotting using anti-*Pf*MCA-2 antibody shown the absence of *Pb*MCA-2 band in Δ*Pb*MCA-2 (c1 and c4) confirming the MCA-2 deletion **(C)**. Immunofluoresence assay depicted the localization of MCA-2 using anti-*Pf*MCA-2 in different stages of the parasite; no FITC-positive parasite was observed in Δ*Pb*MCA-2 (c1 and c4) (upper two panel) compared to wild-type (lower panel) **(D)**.


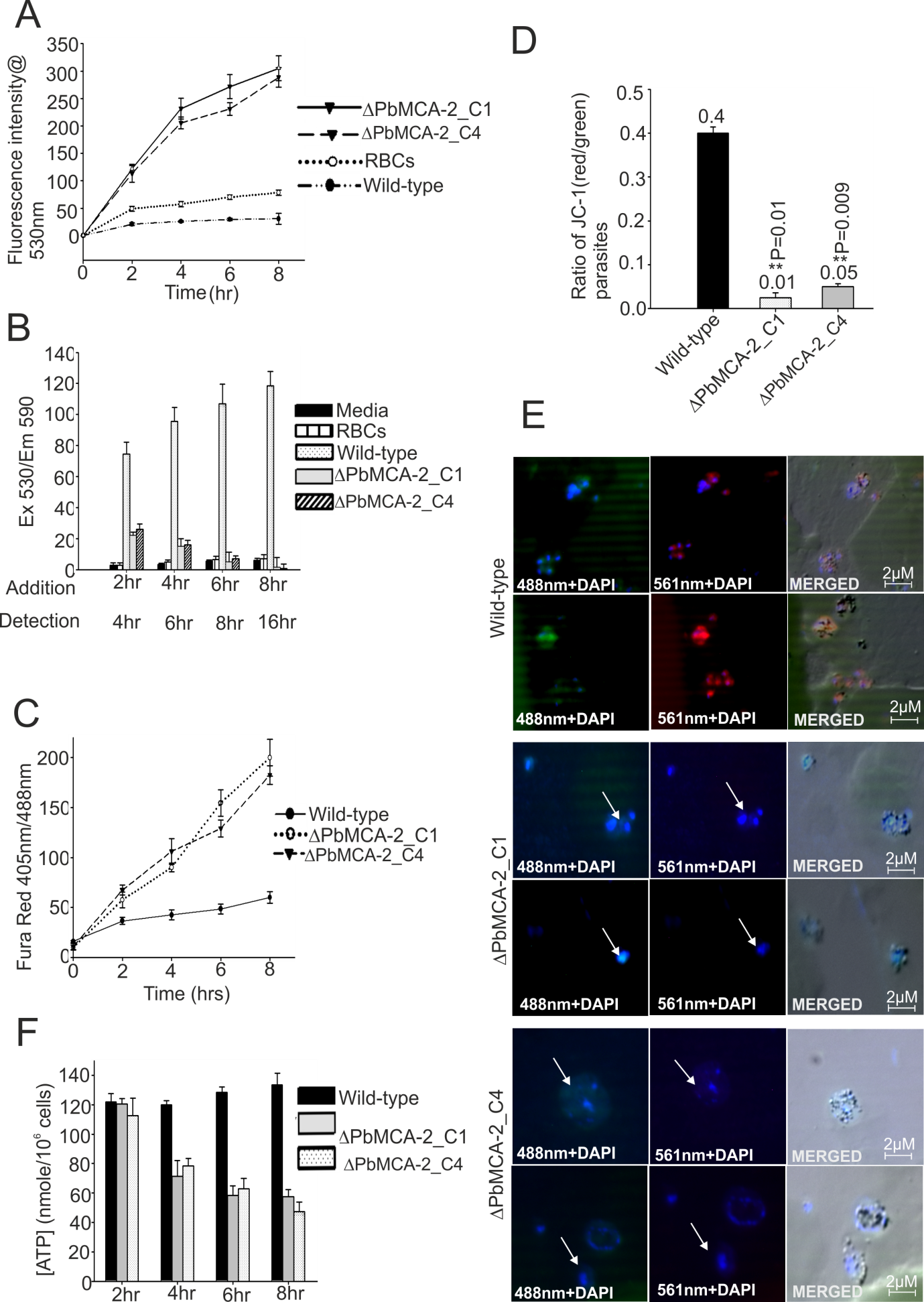


**Supplementary Figure 2:** **Measurement of stress markers in Δ*Pb*MCA-2 parasite line**. ROS level was measured with H2DCFDA dye. After incubation with H2DCFDA, the fluorescence intensity was measured at 530 nm. The values were obtained in triplicates; mean fluorescence was plotted against time **(A)**. Cell viability was determined by using Almar blue dye. AlamarBlue was added at time points, and after addition, fluorescence was measured at different intervals; p < 0.05** (Student's t-test). The values were taken in triplicate and averaged. The fluorescence intensity as a measure of cell viability was plotted against different time points. p< 0.05*(significant); p ≤ 0.01** (highly significant) **(B)**. An increase in Ca^2+^ level was measured in Δ*Pb*MCA-2 parasite line; P=0.01** **(C)**. A bar graph showing the reduction in the ratio of JC-1(red)/JC-1(green) in parasite population of Δ*Pb*MCA-2 parasites; a total of one million cells were counted by flow cytometry to calculate JC-1 ratio; p ≤ 0.01**(highly significant) **(D)**. Fluorescent microscopic images of JC-1-stained representative parasites showing the accumulation of aggregated JC-1(red) in the mitochondria and monomeric JC-1(green) in the cytosol. The parasite nuclei were stained with DAPI (blue) **(E)**. Total cytosolic ATP was measured in Δ*Pb*MCA-2 parasites as described in Materials and methods **(F)**. Data are representative of two independent experiments.


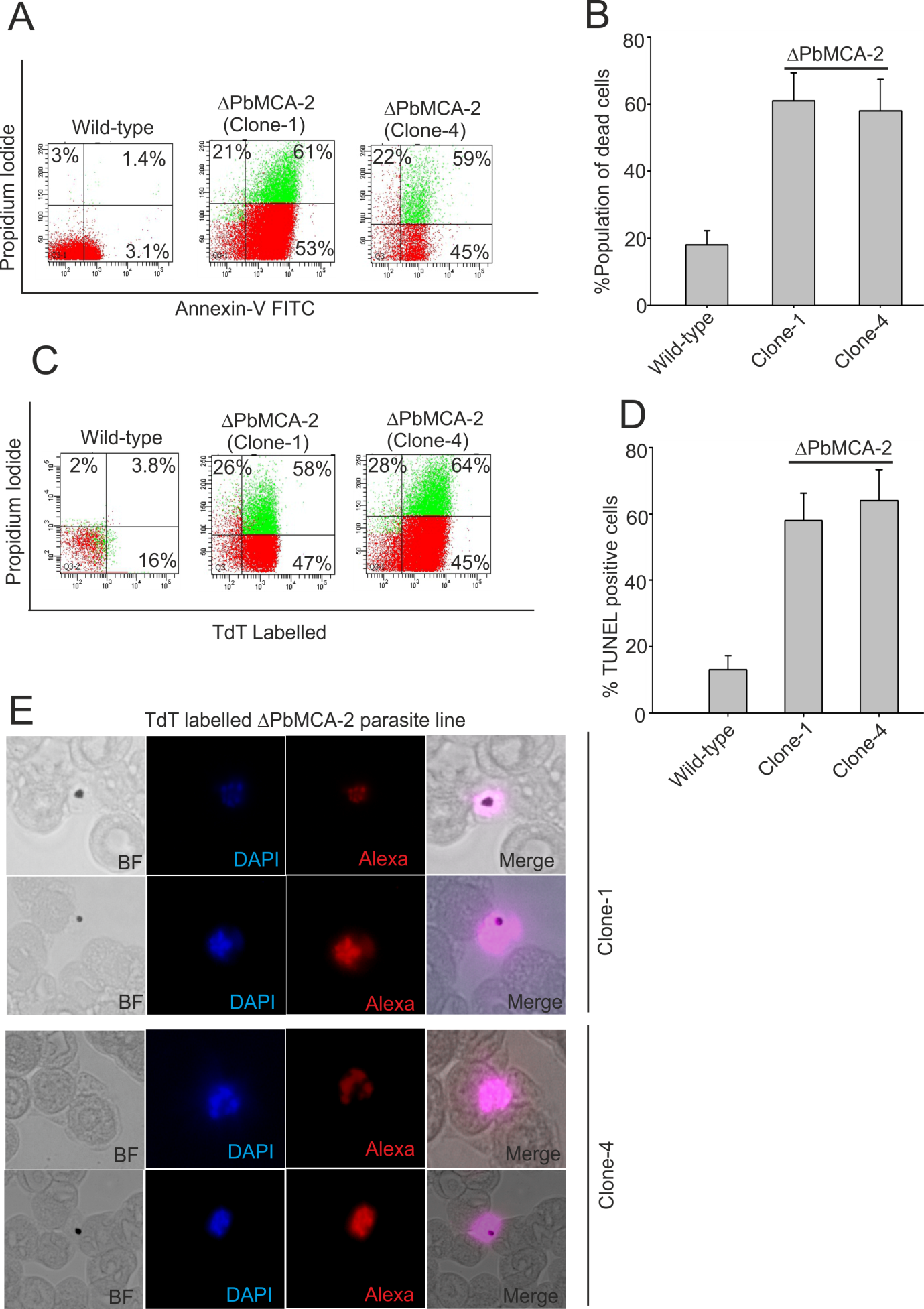


**Supplementary Figure 3**: **Analysis of cell death in Δ*Pb*MCA-2 parasites**. Dot plots represented the population of dead cells analyzed by flow cytometry using annexin-V and propidium iodide in FL-1 versus FL-2 channels, respectively. The cells in the bottom right quadrant indicate apoptosis, whereas cells in the top left quadrant represent the necrotic population **(A)**. Bar graph showed the percentage of dead cells population as determined by FACS analysis **(B)**. Flow cytometry analysis of DNA fragmentation was performed by TdT labelling kit as per the manufacturer's protocol **(C)**. The bar graph represents the percentage of TUNEL-positive cells in Δ*Pb*MCA-2 parasites versus wild-type **(D)**. Images of parasites with fragmented DNA marked as TUNEL-positive cells. Positive cells showed a bright red nucleus. DAPI staining was used to check the location of the nucleus **(E)**. Data are representative of two independent experiments.


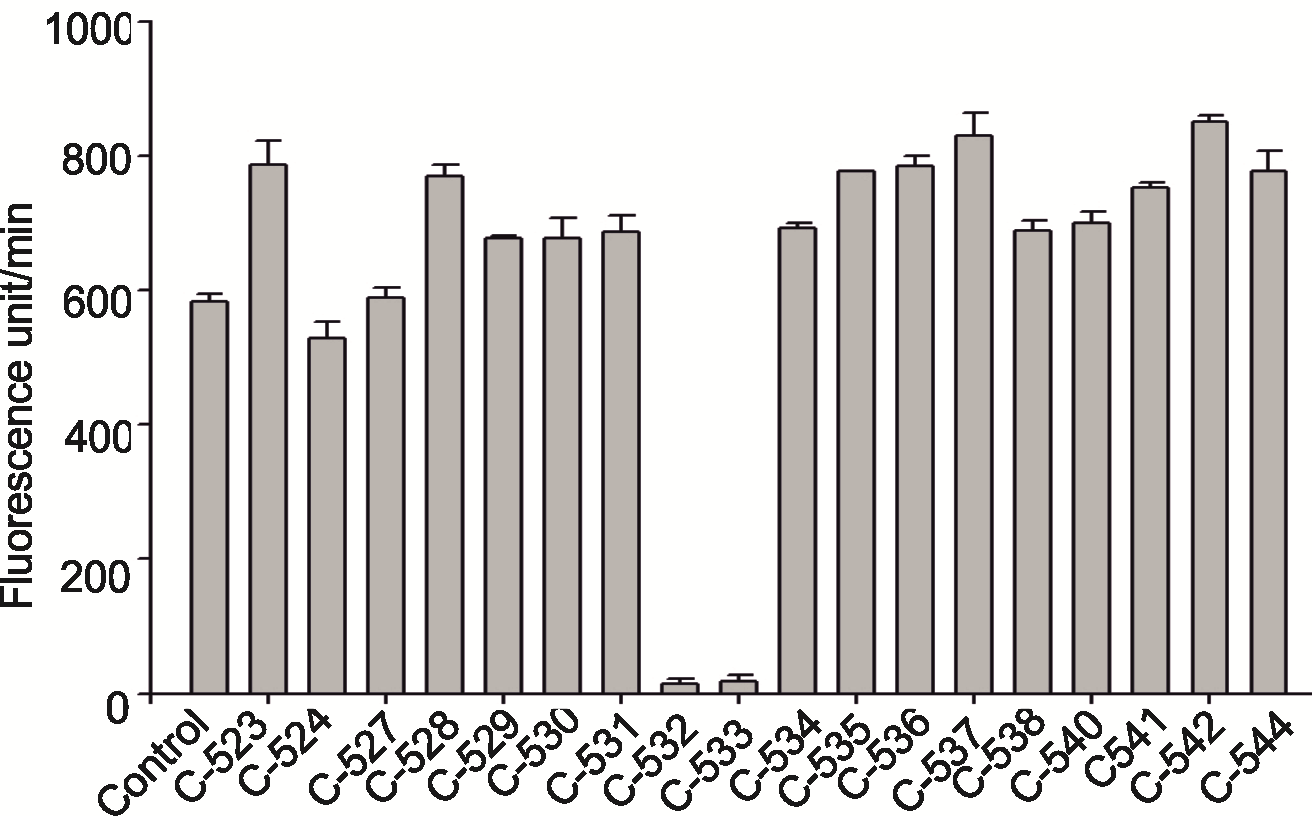


**Supplementary Figure 4.** Enzymatic activity of *Pf*MCA-2 using 50µM of different inhibitors.


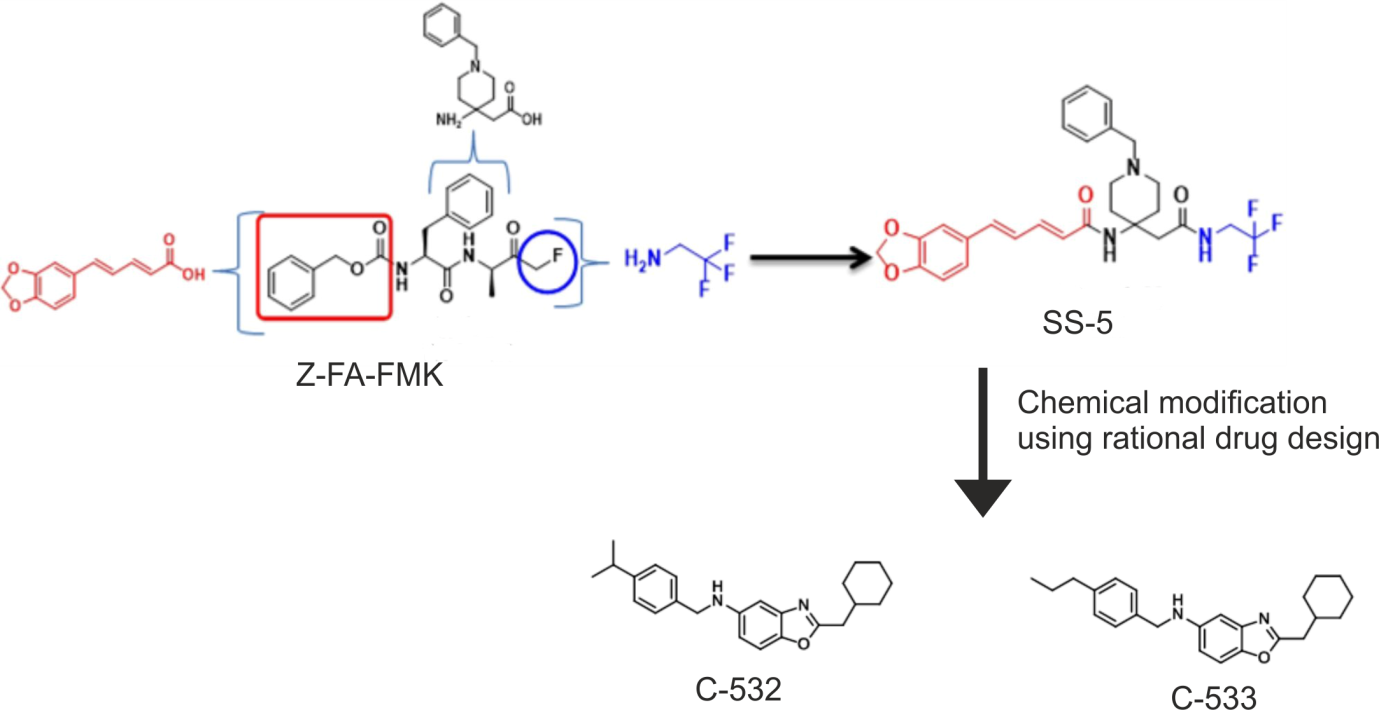


**Supplementary Figure 5**: Synthesis of inhibitory molecules, C-532 and C-533 from parent molecule, SS-5 using rational drug design approach.


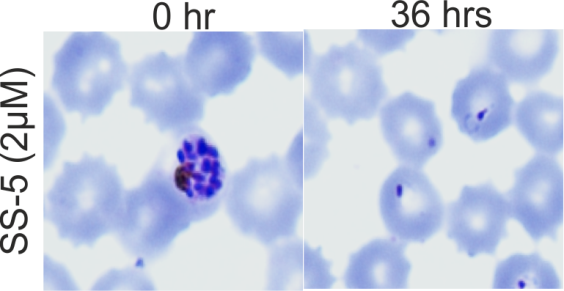


**Supplementary figure 6:** Giemsa stained parasite image showing distorted parasite morphology after treatment with SS-5

**Supplementary Information 1**: Chemical synthesis of MCA-2 inhibitory molecules

**Scheme 1. Reaction conditions:** (a) PPA, 150 ºC, 5 hours (b) H_2_, Pd/C, MeOH, 5 hours (c) (i) Ethanol, 60 ºC, 5-6 hours (ii) Ethanol, NaBH_4_, 0 ºC – rt, 1 hour.

**Synthesis of 2-(cyclohexylmethyl)-5-nitrobenzo[d]oxazole (3):** Commercially available 2-amino-4-nitrophenol (1g, 6.5 mmol), cyclohexylacetic acid (1.02 g, 7.2 mmol) and polyphosphoric acid (PPA; 15 mL) were taken in round bottom flask and heated at 150 ºC for 5 hours. After completion of the reaction, reaction mixture was poured into cursed ice and neutralised by adding dilute NaOH solution. The organic compound was extracted using ethyl acetate and after usual work-up crude organic layer was dried over Na_2_SO_4_ and crude product was purified by column chromatography.

White solid; Yield: 90%; mp 70-72 ºC; IR (KBr, cm^-1^): 2924, 2852, 1618, 1528, 1439, 1347, 1259, 1114, 894, 827, 738, 691; ^1^H NMR (400 MHz, CDCl_3_): δ 1.06-1..33 (m, 5H), 1.66-1.79 (m, 5H), 1.93-2.03 (m, 1H), 2.86 (d, *J* = 7.25 Hz, 2H), 7.58 (d, *J* = 9.00 Hz, 1H), 8.27 (dd, *J* = 8.75, 2.25 Hz, 1H), 8.55 (d, *J* = 2.00 Hz, 1H); ^13^C NMR (100 MHz, CDCl_3_): δ 26.08, 26.17, 33.20, 36.44, 36.69, 110.58, 116.08, 120.77, 142.01, 145.23, 154.50, 170.00.

**Synthesis of compound 2-(cyclohexylmethyl) benzo[d]oxazol-5-amine (4):**

The nitro compound (**3**, 1 g, 3.8 mmol) was dissolved in 15 mL of methanol and shanked for 5 hours in presence of Pd/C (100 mg) catalyst in a paar apparatus at 45 psi pressure of H_2_ gas. After completion of reaction, reaction mixture was filtered through celite pad for removing Pd/C. Filtrate was concentrated using rotavap and the amine obtained was used as such for further reactions.

**Synthesis of compound 5 and 6 :** 2-(cyclohexylmethyl)benzo[d]oxazol-5-amine (300 mg, 1.30 mmol) and desired aldehyde (1.30 mmol) were taken in round bottom flask containing 5 mL of dry ethanol and stirred the reaction mixture for 5-6 hours at 60 ºC. After completion of the reaction, NaBH_4_ (147.42 mg, 3.90 mmol) was added at 0 ºC and continue stirring for 1 hour. Ethanol was removed by using rotavap and ice cold water was added into the reaction mixture. Desired product was extracted using ethyl-acetate solvent and finally concentrated using rotavap. The crude product obtained was purified by using column chromatography and characterised by various analytical techniques.

**2-(cyclohexylmethyl)-N-(4-isopropylbenzyl)benzo[d]oxazol-5-amine (C-532, 5):** White solid; Yield: 75 %; mp 101-103 ºC; IR (KBr, cm^-1^): 3402, 2920, 2849, 1614, 1562, 1485, 1341, 1279, 847, 799, 621, 559, 415; ^1^H NMR (400 MHz, CDCl_3_): δ 1.04-1.28 (m, 11H), 1.64-1.78 (m, 5H), 1.85-1.95 (m, 1H), 2.74 (d, *J* = 6.87 Hz, 2H), 2.87-2.94 (m, 1H), 4.04 (brs, 1H), 4.30 (s, 2H), 6.61 (dd, *J* = 8.70, 2.29 Hz, 1H), 6.88 (d, *J* = 1.83 Hz, 1H), 7.20-7.32 (m, 5H); ^13^C NMR (100 MHz, CDCl_3_): δ 24.16, 26.14, 26.27, 33.19, 33.94, 36.53, 36.78, 49.01, 101.89, 110.44, 111.51, 126.83, 127.72, 136.66, 142.62, 144.16, 145.91, 148.09, 166.97; ESI-HRMS (m/z) calcd. for C_24_H_30_N_2_O: 362.2358; Found: 363.2392 (MH)^+^.

**2-(cyclohexylmethyl)-N-(4-propylbenzyl)benzo[d]oxazol-5-amine (C-533):** Brownish solid; Yield: 74 %; mp 72-74 ºC; IR (KBr, cm^-1^): 3325, 2922, 2849, 1618, 1560, 1489, 1443, 1350, 1290, 1200, 1173, 968, 831, 800, 629, 467, 428, 417; ^1^H NMR (400 MHz, CDCl_3_): δ 0.94 (t, *J* = 7.33 Hz, 3H), 1.01-1.31 (m, 5H), 1.58-1.77 (m, 7H), 1.89-1.94 (m, 1H), 2.57 (t, *J* = 8.24 Hz, 2H), 2.74 (d, *J* = 6.87 Hz, 2H), 4.05 (brs, 1H), 4.30 (s, 2H), 6.60 (dd, *J* = 8.70, 2.29 Hz, 1H), 6.88 (d, *J* = 1.83 Hz, 1H), 7.14-7.30 (m, 5H); ^13^C NMR (100 MHz, CDCl_3_): δ 13.99, 24.73, 26.16, 26.29, 33.22, 36.54, 36.80, 37.85, 49.09, 101.99, 110.44, 111.53, 127.66, 128.88, 136.54, 141.93, 142.64, 144.21, 145.90, 166.98; ESI-HRMS (m/z) calcd. for C_24_H_30_N_2_O: 362.2358; Found: 363.2378 (MH)^+^.


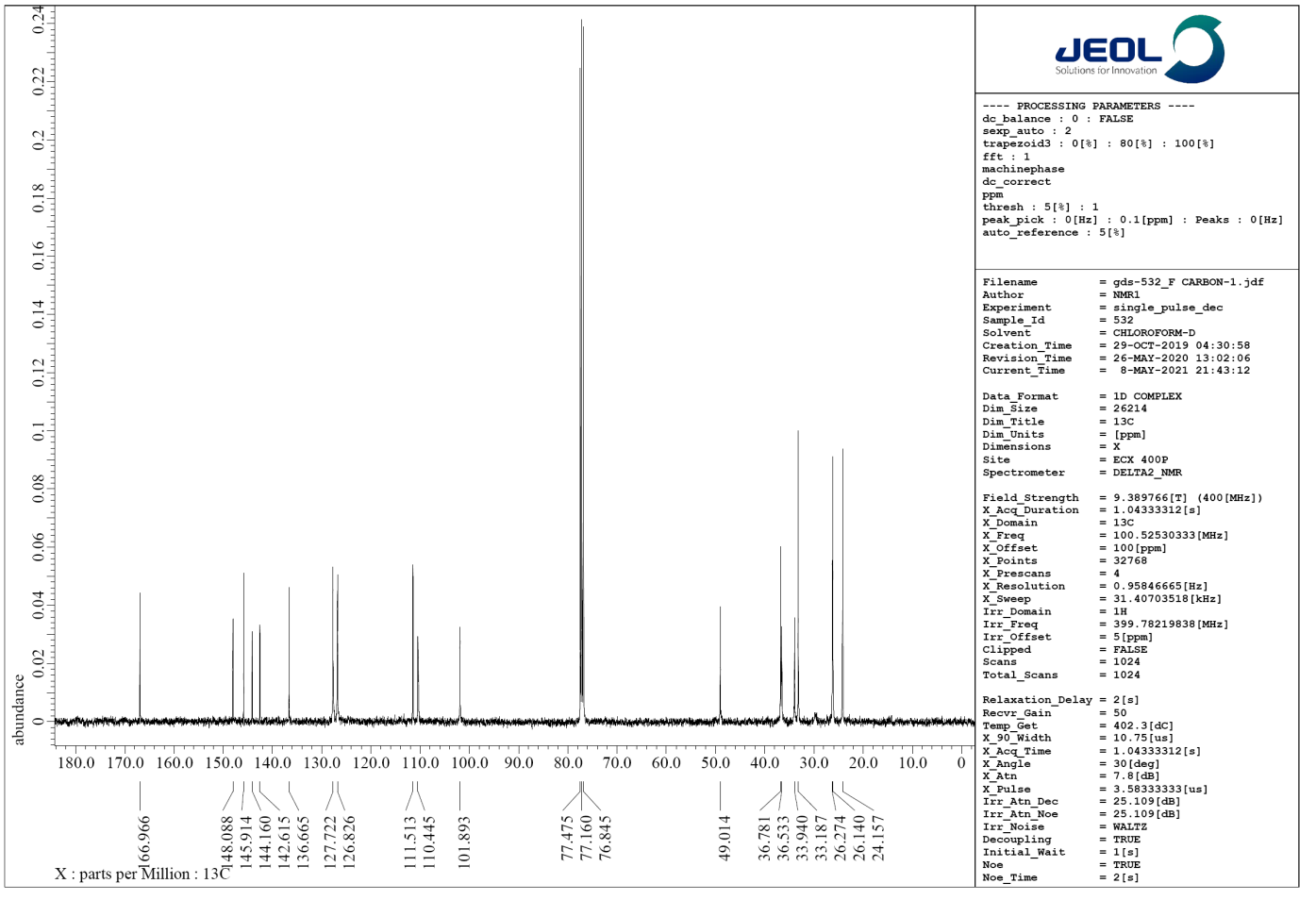


^1^H and ^13^C NMR of C-532


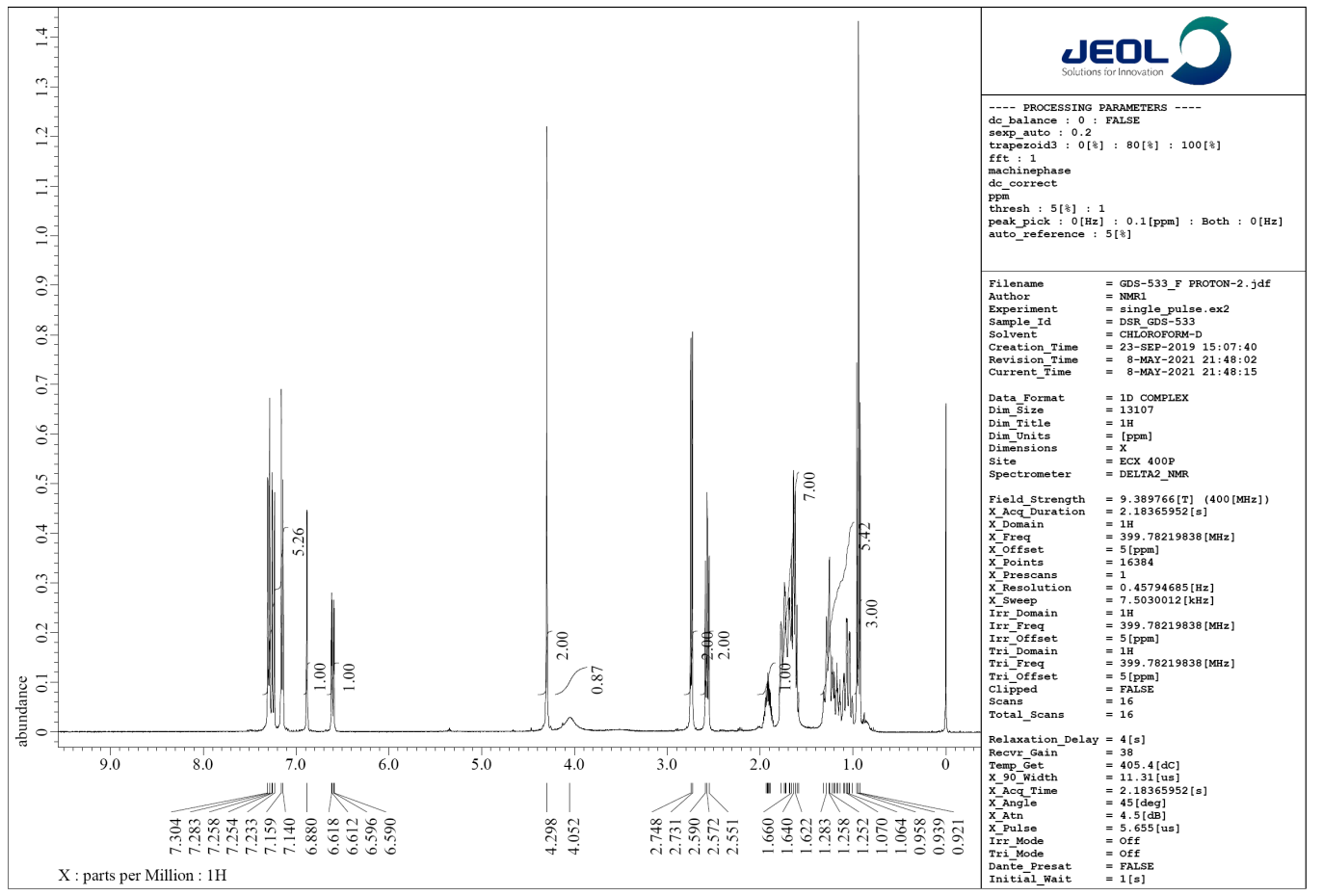


^1^H and ^13^C NMR of C-533

**Table 1 List of primers used in the study**

| **S. No** | **Name** | **Primer sequence** |
| --- | --- | --- |
| 1 | MCA-2_5’UTR | Fw- **5’**ATGCCTCGAGGGTCGGATTTTAACAATTGTATG**’3**  Rev- **3’**GTACATCGATGTGGTATTCACTGAATACACA**’5** |
| 2 | MCA-2_3’UTR | Fw- **5’**TGATCAGAGCGGCCGCTATGACTATGAATGTTCATA GCT**’3**  Rev- **3’**TGATGGCGCGCCTCATAGATACATTGTTGAAACGGT**’5** |
| 3 | DHFR | Fw- **5’**GTTGTCTCTTCAATGATTCAT**’3** |

**Table 2.** Response to treatment of different antimalarial compounds in BALBc mice infected with *P*. berghei on day 8.

| **Dose of compound** | **% Parasitaemia ± SD** | **% Parasite suppression ± SD** |
| --- | --- | --- |
| Artemisinin  10mg/kg | 0.3±0.05 | 88±2 |
| C-532 (10mg/kg) | 0.05±0.02 | ~95 |
| C-533  (10mg/kg) | 0.03±0.05 | ~92 |

**Table 3 Infectivity by biological transmission or with *P. berghei* sporozoites injected into C57BL/6 mice, and pre-patent period was determined by microscopy.**

| **S. No** | **Parasites** | **Route** | **Sporozoites** | **Mice positive/mice inoculated** | **Pre-patent period (Day)** |
| --- | --- | --- | --- | --- | --- |
| 1 | Wild | Mosquito bite | From 10 mosquito | 6/6 | 4 |
|  | Δ*Pb*MCA-2 (Clone-2 | Mosquito bite | From 10 mosquito | 4/4 | 6 |
|  | Δ*Pb*MCA-2 (Clone-4 | Mosquito bite | From 10 mosquito | 4/4 | 6 |
